# Different resources for different times: sense of coherence and emotional intelligence as correlates of adaptation in six cohorts of medical students

**DOI:** 10.3389/fmed.2026.1860440

**Published:** 2026-06-23

**Authors:** Maciej Walkiewicz

**Affiliations:** Department of Psychology, Medical University of Gdańsk, Gdańsk, Poland

**Keywords:** adaptation, cross-cohort comparison, emotional intelligence, medical education, medical students, restriction of range, salutogenesis, sense of coherence

## Abstract

**Background:**

Sense of coherence (SOC) and emotional intelligence (EI) are both conceptualised as psychological resources protecting medical students from the demands of their training. Whether their associations with indicators of adaptation are stable across cohorts recruited at different time points has not previously been examined within a single multi-cohort study at one institution.

**Methods:**

We analysed data from six independent cross-cohort of second-year medical students at the Medical University of Gdańsk, Poland, between 2014 and 2024 (*N* = 1,595; 64% female; mean age 20.97, SD 1.52). SOC was measured with the 29-item Life Orientation Questionnaire (SOC-29) and EI with the Polish INTE (adaptation of the Schutte Self-Report Emotional Intelligence Test). Adaptation was indexed by general life evaluation (quality of life, 6-point scale), current well-being (4-point scale), academic stress (visual analogue scale 1–10), and satisfaction with medical studies (1–10).

**Results:**

In the 2014 cohort, SOC showed a clear gradient with quality of life (mean difference “wonderful” vs. “not very good” = 48.8 points, 95% CI: 26.0–71.6); no comparable gradient was detected in the 2015–2024 cohorts. Total EI was highest in the 2024 cohort (Md = 124) compared with 2015–2018 (Md = 118–119) and showed gradients with adaptation indicators in several cohorts. The SOC variance in 2015–2018 substantially reduces statistical power to detect associations in these cohorts.

**Conclusion:**

SOC and EI showed different patterns of association with indicators of adaptation across the six cohorts examined. This implies a need to shift the emphasis in mentoring programmes from building a global sense of meaning toward developing specific emotional competencies.

## 1 Introduction

Medical students worldwide experience high levels of psychological distress encompassing professional burnout, workforce attrition, and negatively impacting the quality of patient care (1–3). Identifying internal psychological resources that protect against maladaptation is therefore a research priority in medical education. Two constructs have attracted particular attention in this context: sense of coherence (SOC), rooted in Antonovsky’s salutogenic theory (4, 5), and emotional intelligence (EI) (6, 7). Although both are conceptualised as resources supporting coping with the demands of the clinical environment, they have not previously been examined together within a single multi-cohort study. Nor has the question been raised whether their protective role is stable and universal or whether it shifts across successive generations of students. Furthermore, conducting such a study in Poland, a region that has experienced rapid socio-educational transformations and recent geopolitical crises, provides a unique backdrop for examining these dynamics.

SOC, defined as a global orientation reflecting a sense of comprehensibility, manageability, and meaningfulness of life experiences (5), is regularly described as a psychological resource in medical education. It has been linked to academic stress (8, 9), professional burnout (10), and psychological well-being (11, 12). In terms of long-term outcomes, a 10-year longitudinal study conducted on the same medical student population demonstrated that SOC measured at admission differentiated styles of career success 4 years after graduation, with the highest-SOC students achieving the highest quality of life and income yet the lowest career satisfaction and professional competence (13, 14). These findings suggest that SOC is not a straightforward predictor of “success” but rather a predictor of a specific profile of professional functioning. However, existing research relies almost exclusively on single cohorts, making it impossible to assess whether the protective role of SOC is stable across successive generations of students or changes over time.

In parallel, interest has grown in EI as an adaptive resource in medical education (6, 15). EI, understood as the ability to recognise, understand, and regulate one’s own emotions (the intrapersonal component) and those of others (the interpersonal component), is increasingly studied in the context of Professional Identity Formation (PIF), clinical empathy, and academic success (7, 16, 17). The most recent AMEE review emphasises that EI constitutes a fundamental pillar of physicians’ professional identity development (7).

Within Antonovsky’s salutogenic model (5), Generalised Resistance Resources (GRRs) encompass all factors that facilitate coping with stressors and strengthen SOC. EI, as a capacity for emotional regulation, meets the criteria for a GRR (18). A key empirical question arises: if EI is a GRR that strengthens SOC, should both resources exhibit parallel cross-cohort trends, or can they diverge in ways that would raise questions for the hierarchical GRR → SOC model?

A critical gap in the literature is the absence of studies examining SOC and EI side by side in the same population across a multi-cohort design. This precludes comparison of their patterns of association with adaptation and assessment of whether both constructs follow similar or divergent generational trends. There are theoretical grounds to expect that the two constructs may respond differently to generational change. SOC is conceptualised as a relatively stable life orientation shaped during early adulthood (5, 19), whereas EI is considered more malleable and amenable to training (6, 15). Socio-cultural changes of recent decades, digitalisation, the evolution of emotional socialisation patterns, the COVID-19 pandemic, and medical curriculum reforms may affect the two constructs to different degrees. If resistance resources show cross-cohort variability, this would suggest that the salutogenic framework in medical education could be examined further with regard to cohort dynamics, although such inferences require longitudinal designs and measurement invariance testing beyond the scope of the present cross-cohort study.

The aim of the present study, encompassing six cohorts of medical students (2014–2024, *N* = 1,595), was to describe the cross-cohort associations between SOC, EI, and indicators of adaptation to medical studies, and to compare these associations across six independent cohorts. The following hypotheses were tested: (1) both SOC and EI are significantly associated with indicators of adaptation to medical studies; (2) the strength of the SOC-adaptation association differs across cohorts; (3) EI shows a more consistent cross-cohort association with adaptation than SOC, with gradients present in a greater number of cohorts; and (4) the cross-cohort trends of SOC and EI differ in direction.

## 2 Materials and methods

### 2.1 Study design

A cross-cohort comparison design was employed. Data collected at selected time points between 2014 and 2024 were analysed. The aim was not continuous monitoring of change but rather a comparison of the levels of the studied variables in comparable groups of second-year medical students assessed under different social and educational contexts. The data originated from two distinct periods within a long-term research programme conducted at the Medical University of Gdańsk:

2014–2018: five independent cohorts assessed as part of a project on student well-being and adaptation.

2024: the first post-pandemic measurement, conducted after the stabilisation of teaching conditions.

The irregular intervals between measurements resulted from the opportunistic nature of participant availability and organisational constraints. The gap in data collection between 2020 and 2023 was attributable to two factors: (1) in 2020–2021, the COVID-19 pandemic precluded conducting research under standard on-site conditions; (2) in 2022–2023, the priority was stabilising the teaching process, which limited the scope for undertaking additional research projects. The 2024 measurement was conducted after a full return to pre-pandemic conditions of study organisation.

The choice of second-year students was guided by three methodological considerations: (1) this period represents a phase of relative adaptive stabilisation following the intensive stress of the first year; (2) it precedes the stage of full clinical workload and patient responsibility; and (3) the curriculum at this stage ensures the greatest comparability across the analysed cohorts over a 10-year span. These factors allow adaptation to be assessed as a relatively stable psychological resource, with limited influence from short-term situational factors and varied clinical experiences.

### 2.2 Participants

The study included a total of *N* = 1,595 s-year medical students at the Medical University of Gdańsk. Recruitment was carried out through student group representatives and announcements during psychology classes. Participation was voluntary and anonymous. To minimise social desirability bias, data collection was administered by individuals independent of the research team. All measurements were conducted during April–May, enabling control for the effect of the phase of the academic year.

Inclusion criteria were: second-year medical student status and fluency in Polish. Exclusion criteria were: insufficient Polish language proficiency or enrolment in a year other than the second at the time of the study.

### 2.3 Measures

#### 2.3.1 Sense of coherence (SOC)

The sense of coherence was measured using the 29-item Life Orientation Questionnaire (SOC-29; 5). The instrument consists of 29 items. It enables the assessment of the overall level of sense of coherence as well as its three components: comprehensibility (11 items), manageability (10 items), and meaningfulness (8 items). Responses are provided on a 7-point semantic differential scale, with total scores ranging from 29 to 203 points. Higher values indicate a stronger sense of coherence. An identical Polish version of the SOC-29 questionnaire was administered across all cohorts following a standardized procedure, ensuring measurement equivalence between time points. The instrument is characterised by robust psychometric properties; Cronbach’s alpha coefficient in populations of students and healthcare professionals typically ranges from 0.82 to 0.95 (12).

#### 2.3.2 Emotional intelligence (EI)

Emotional intelligence (EI) was measured using the Polish adaptation of the Schutte Self-Report Emotional Intelligence Test [SSEIT; (20)]-the Emotional Intelligence Questionnaire (INTE), developed by Jaworowska and Matczak (21). The instrument is grounded in the theoretical model of EI proposed by Salovey and Mayer (22), which defines EI as the ability to recognise, understand, and regulate one’s own and others’ emotions, as well as to effectively use emotions to guide thinking and behaviour. The questionnaire consists of 33 self-report items rated on a 5-point Likert scale (from 1 = “strongly disagree” to 5 = “strongly agree”). The Polish version yields a total score and two factor-based subscales: interpersonal EI (the ability to recognise emotions in others) and intrapersonal EI (the ability to utilise one’s own emotions). The Polish adaptation of the INTE demonstrates high internal consistency (Cronbach’s *α* = 0.87) and satisfactory temporal stability (21).

#### 2.3.3 Dependent variables: indicators of adaptation

In this study, adaptation to medical studies is conceptualised within Antonovsky’s salutogenic ease/dis-ease continuum (4, 5), reflecting an individual’s adaptive position along that continuum in the specific context of medical education. While the indicators chosen overlap conceptually with subjective well-being and quality of life, in the present framework they jointly operationalise adaptation as a multidimensional construct combining global life evaluation, current well-being, context-specific stress, and context-specific satisfaction. Adaptation was operationalised through four indicators:

1. Quality of life (general life evaluation): participants rated their life to date on a 6-point categorical scale ranging from “unhappy” to “wonderful.”
2. Current well-being: Participants evaluated their quality of life over the past 2 weeks on a 4-point scale (from “unhappy” to “very happy”).
3. Academic stress: Stress levels related specifically to medical education were measured using a Visual Analogue Scale (VAS) from 1 (no stress) to 10 (extreme stress).
4. Satisfaction with medical studies: Students rated their overall satisfaction with studying medicine on a scale from 1 to 10. Single-item global indicators of life evaluation, well-being, academic stress, and study satisfaction were chosen over longer validated instruments (e.g., WHOQOL) to maintain identical measurement across all six cohorts spanning 10 years; switching instruments in the most recent cohort would have compromised cross-cohort comparability, which is the central design feature of this study. Single-item evaluations of life and well-being have demonstrated acceptable reliability and validity in adult populations; the absence of multi-item validated instruments is acknowledged in section 4.1.

### 2.4 Statistical analysis

Due to the non-normal distribution of scores in the majority of analysed subgroups, between-group comparisons were conducted using the Kruskal-Wallis test with Dunn’s post-hoc tests (Benjamini-Hochberg correction). To examine the interaction between cohort (year) and adaptation indicators, two-way analysis of variance (Type III ANOVA) with Games-Howell post-hoc tests was employed. The use of a parametric approach for ANOVA was justified by the normality of distributions within subgroups (after simultaneous partitioning by cohort year and adaptation categories) and the satisfaction of the homogeneity of variance assumption. Categories with fewer than 15 observations were excluded from the ANOVA analyses. The significance level was set at *α* = 0.05. All statistical analyses were performed using R software (version 4.4.1). The combined use of non-parametric and parametric tests reflects different distributional contexts: pooled distributions across categories were skewed, motivating Kruskal-Wallis with Dunn post-hoc and Benjamini-Hochberg correction for between-group comparisons; within finer subgroups (cohort × adaptation category), distributions were approximately normal, supporting two-way ANOVA. Games-Howell post-hoc was used to accommodate possible heterogeneity of variance across subgroups. Given the number of interaction tests across four adaptation indicators and multiple constructs, isolated significant post-hoc results—particularly directional reversals (e.g., in the 2016 and 2017 subgroups), should be interpreted with caution and are flagged as candidates for Type I error in the Discussion. Effect-size estimates for between-group comparisons and ANOVA contrasts will be added in revised Supplementary Tables. To address concerns about whether large samples may yield statistically significant but practically negligible differences, Cohen’s d effect-size estimates have been added to Supplementary Tables S3, S6, S9, S18, S20, and S23. Across the 48 significant ANOVA post-hoc contrasts, |d| ranged from 0.46 to 2.99 (median 1.11), indicating predominantly large to very large practical effects (38 of 48 contrasts had |d| ≥ 0.80). Effect sizes were computed using the simple pooled-SD formula d = (M₁ − M₂)/√[(SD₁^2^ + SD₂^2^)/2] directly from the means and standard deviations reported in the descriptive tables.

## 3 Results

Full statistical tables, descriptive statistics by cohort and adaptation category, and complete post-hoc comparisons are provided in Supplementary Tables S1–S28 (Appendix 1). Three figures and three tables summarise the key findings.

Characteristics of the individual cohorts are presented in Table 1.

**Table 1:** Participant characteristics by cohort (*N* = 1,595).

Cohort	(*N*) Eligible	*n* (Response rate)	% total of 1,595	Female	Male	Age M (SD)
2014	337	297 (88%)	19	67%	33%	20.98 (1.57)
2015	255	236 (92%)	15	67%	33%	20.65 (1.10)
2016	252	214 (84%)	13	62%	38%	20.64 (1.56)
2017	292	284 (97%)	19	59%	41%	21.01 (1.39)
2018	310	270 (87%)	17	66%	33%	20.96 (1.39)
2024	445	294 (66%)	18	65%	35%	21.45 (1.83)

Due to missing data in individual questionnaires, effective sample sizes in specific analyses differ slightly from the total number of participants in a given cohort. For EI analyses, the effective samples were: 2016 *n* = 205, 2024 *n* = 262 (Supplementary Table S11).

### 3.1 Sense of coherence and adaptation to medical studies

#### 3.1.1 Inter-cohort differences in SOC levels

A Kruskal-Wallis test revealed statistically significant differences in SOC levels across cohorts (*p* < 0.001). Median SOC was highest in the 2014 cohort (Md = 129, Q1 = 117, Q3 = 143) and systematically lower in subsequent cohorts: 2015 (Md = 102, Q1 = 97, Q3 = 108), 2016 (Md = 103, Q1 = 97, Q3 = 110), 2017 (Md = 103, Q1 = 97, Q3 = 109), 2018 (Md = 105, Q1 = 98, Q3 = 111), and 2024 (Md = 101, Q1 = 93, Q3 = 112).

Post-hoc tests (Dunn with Benjamini-Hochberg correction) confirmed that the 2014 cohort had significantly higher SOC than each of the remaining cohorts (all *p* < 0.05). None of the pairwise comparisons among the 2015–2024 cohorts reached significance. Full rank statistics are reported in the Appendix 1.

**Figure 1:**
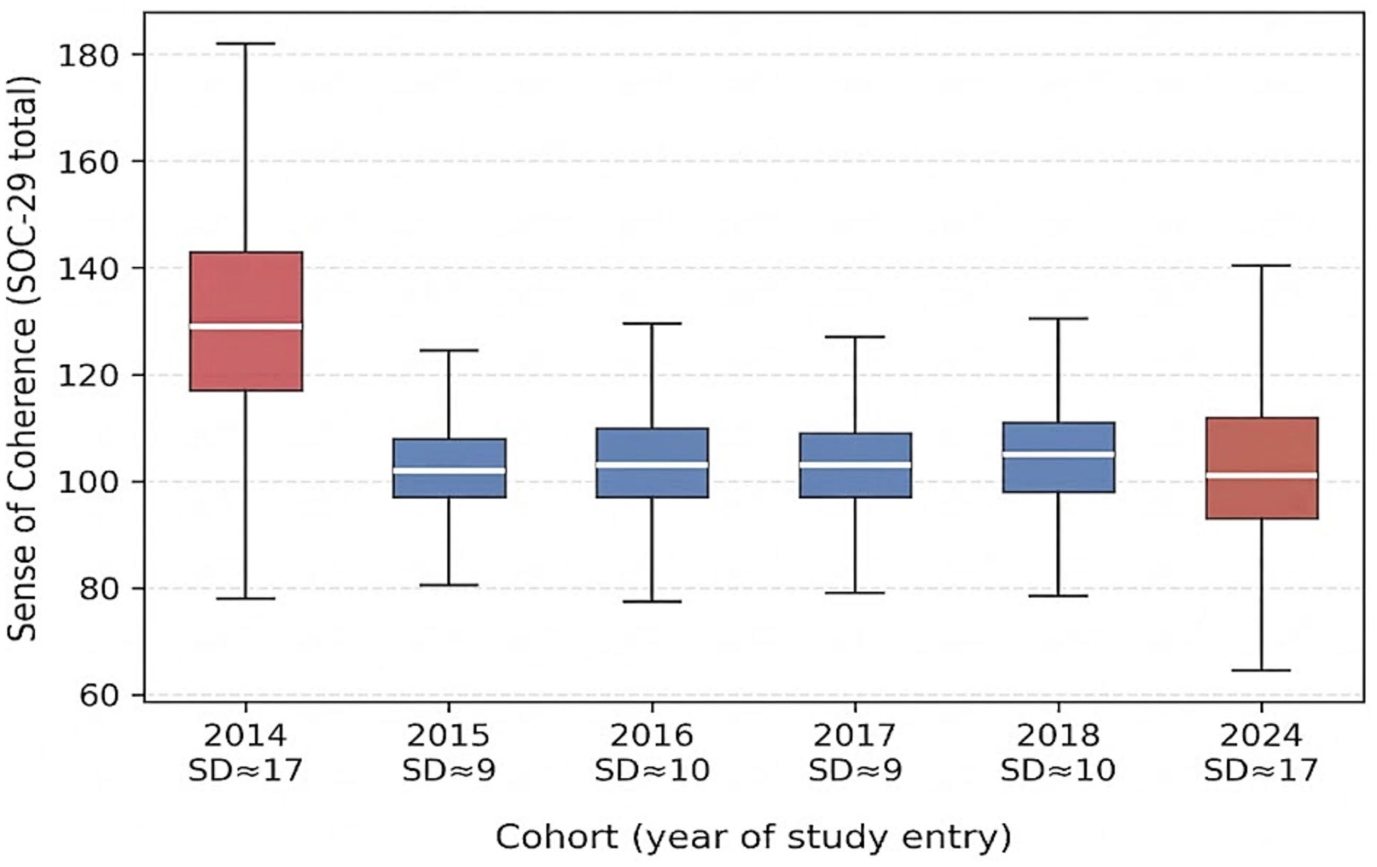
Distributions of sense of coherence (SOC-29 total) across six cohorts of second-year medical students. Boxes show the interquartile range (IQR); the white horizontal line within each box shows the median; whiskers extend to Q1–1.5 × IQR and Q3 + 1.5 × IQR. The 2014 and 2024 cohorts (red) exhibit markedly greater dispersion than the 2015–2018 cohorts (blue). This variance heterogeneity is a critical interpretive factor for the cross-cohort comparison (see Discussion).

Notably, the 2014 cohort was characterised not only by a higher median but also by substantially greater score dispersion (SD ≈ 16–19 depending on subgroup) compared with the 2015–2018 cohorts (SD ≈ 7–11), while the 2024 cohort again showed increased variability (SD ≈ 15–21). This dramatic variance difference constitutes an important interpretive factor: restriction of range in the 2015–2018 cohorts may have attenuated the detectability of SOC-adaptation associations in those cohorts (see Discussion) (see Figure 1).

#### 3.1.2 SOC and adaptation indicators-overall analysis

In the overall analysis (*N* = 1,595), SOC showed limited associations with adaptation indicators: satisfaction with medical studies (*p* = 0.029), with one significant post-hoc difference (satisfaction = 1 vs. satisfaction = 3; rank 939 vs. 582, *p* = 0.041); quality of life (*p* = 0.090), borderline; well-being (*p* = 0.072), not significant; and academic stress (*p* = 0.700), clearly not significant.

#### 3.1.3 Cohort × adaptation interaction for SOC

Two-way ANOVA (Type III) revealed significant interactions between cohort and adaptation indicators, indicating that the SOC-adaptation association was moderated by cohort year.

SOC × Quality of life. Main effect of cohort: *F*(1) = 24.44, *p* < 0.0001. Main effect of QOL: *F*(4) = 2.58, *p* = 0.036. Cohort × QOL interaction: *F*(4) = 2.58, *p* = 0.036. Games-Howell post-hoc tests showed that the SOC gradient across life evaluation categories was pronounced exclusively in the 2014 cohort: “wonderful” M = 142.8 (SD = 17.0) versus “not very good” M = 94.0 (SD = 15.6); difference = −48.8 points (95% CI: −71.6 to −26.0), *p* < 0.0001. Differences between “wonderful” and “neither good nor bad” (−28.7; *p* < 0.001), “quite good” (−16.8; *p* < 0.001), and “successful” (−12.1; *p* = 0.001) were likewise significant only in 2014.

In the 2016 cohort, a reversed pattern was observed: students rating their life as “wonderful” had significantly lower SOC than those responding “neither good nor bad” (M = 100.0, SD = 9.4 vs. M = 109.1, SD = 10.6; difference = 9.1, 95% CI: 1.4 to 16.8; *p* = 0.012). No significant post-hoc differences were found in the 2015, 2017, 2018, or 2024 cohorts.

SOC × Academic stress. Main effect of cohort: *F*(1) = 119.28, *p* < 0.0001. Main effect of academic stress: *F*(7) = 2.15, *p* = 0.036. Cohort × academic stress interaction: *F*(7) = 2.14, *p* = 0.037. Significant post-hoc differences were found exclusively in the 2014 cohort, where lower stress was associated with higher SOC: stress = 3 (M = 150.9) versus stress = 9 (M = 121.1); difference = −29.8 (95% CI: −46.3 to −13.3), *p* < 0.0001. No significant differences were observed in the 2015–2024 cohorts.

SOC × Satisfaction with medical studies. Main effect of cohort: *F*(1) = 35.85, p < 0.0001. Main effect of satisfaction: *F*(7) = 2.03, *p* = 0.049. Cohort × satisfaction interaction: *F*(7) = 2.02, *p* = 0.050. Both the main effect of satisfaction and the interaction were at the boundary of conventional significance. Isolated significant comparisons emerged: in the 2014 cohort, satisfaction = 9 (M = 135.8) versus satisfaction = 6 (M = 121.2), *p* = 0.042; in the 2017 cohort, satisfaction = 1 (M = 116.0) versus satisfaction = 8 (M = 100.5), *p* = 0.005. Of note, the latter result represents a reversal of the expected direction, students with the lowest satisfaction had paradoxically higher SOC than those with moderately high satisfaction.

SOC × Well-being. Cohort × well-being interaction: *F*(3) = 0.37, *p* = 0.777, not significant. The only significant effect was the main effect of cohort (*F* = 67.40, *p* < 0.0001).

Full descriptive statistics and ANOVA tables for all SOC analyses are presented in Supplementary Tables S1–S10.

#### 3.1.4 Summary of the SOC pattern

Sense of coherence showed a strong cohort effect: the 2014 cohort had dramatically higher SOC than all others. The SOC-adaptation association took the form of a monotonic gradient exclusively in this cohort. In the 2015–2024 cohorts, the differentiating role of SOC was minimal or absent. A reversal of the effect direction was observed in the 2016 cohort. Hypothesis H2, moderation by cohort, was confirmed.

### 3.2 Emotional intelligence and adaptation to medical studies

#### 3.2.1 Total EI-inter-cohort differences

**Figure 2:**
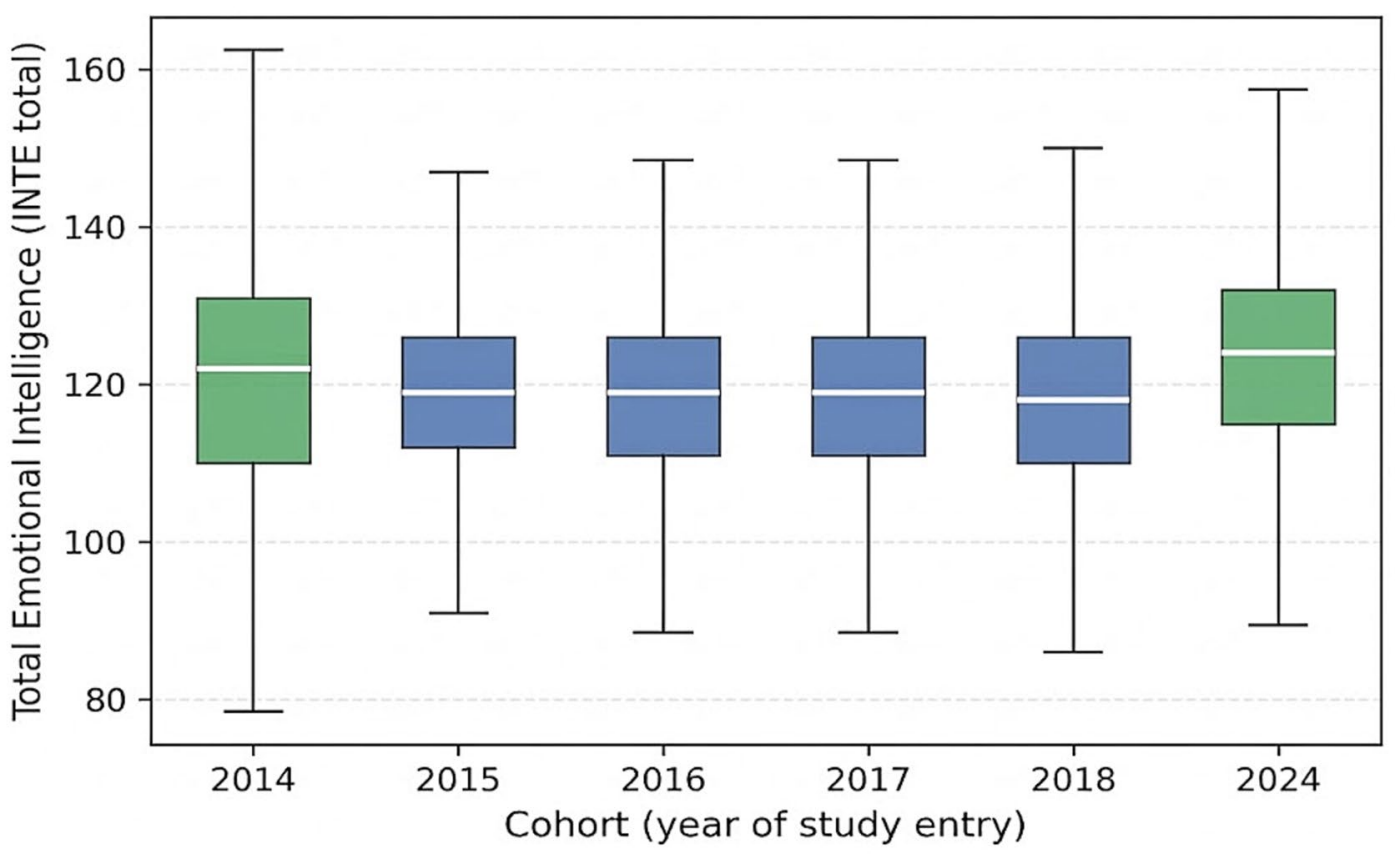
Distributions of total emotional intelligence (INTE total) across six cohorts of second-year medical students. Boxes show the interquartile range; the white line within each box shows the median; whiskers extend 1.5 × IQR beyond Q1/Q3. The 2014 and 2024 cohorts (green) showed the highest medians; in contrast to SOC (Figure 1), EI variance does not compress in the 2015–2018 cohorts.

A Kruskal-Wallis test revealed significant differences in total EI across cohorts (*p* < 0.001). In contrast to SOC, the 2024 cohort had the highest median EI (Md = 124, Q1 = 115, Q3 = 132), significantly higher than the 2015–2018 cohorts (Md = 118–119; all *p* ≤ 0.001; Supplementary Table S12). The 2014 cohort (Md = 122) did not differ significantly from the 2024 cohort (see Figure 2).

#### 3.2.2 Total EI and adaptation indicators-overall analysis

In contrast to SOC, total EI showed strong, consistent associations with all adaptation indicators:

Quality of life (QOL): *p* < 0.001. A clear monotonic gradient was observed, each higher life evaluation category was associated with significantly higher EI. Students rating their life as “wonderful” differed significantly from all lower categories (all *p* ≤ 0.002), and the gradient was hierarchical, with significant differences between adjacent categories (full post-hoc results: Supplementary Table S13).

Well-being: *p* < 0.001. An ordered, complete four-level gradient, each successive well-being category differed significantly from the preceding one (all *p* ≤ 0.043; Supplementary Table S14).

Satisfaction with medical studies: p < 0.001. The most pronounced gradient among all measures. Median EI increased monotonically from satisfaction = 1 (Md = 99) to satisfaction = 10 (Md = 127). Significant post-hoc comparisons spanned a wide range of pairs, from the lowest to the highest satisfaction levels (full list: Supplementary Table S16).

Academic stress: *p* = 0.004. A weaker association, significant differences were limited to the comparisons stress = 3 versus stress = 8 (*p* = 0.002) and stress = 3 versus stress = 9 (*p* = 0.022).

#### 3.2.3 Cohort × adaptation interaction for total EI

In a key contrast with SOC, the ANOVA revealed that the EI-adaptation gradient was present across multiple cohorts, not only in 2014:

EI × QOL × cohort. Main effect of cohort: *F*(1) = 16.96, *p* < 0.0001. Main effect of QOL: *F*(4) = 6.36, *p* < 0.0001. Cohort × QOL interaction: *F*(4) = 6.32, *p* < 0.0001. Post-hoc tests revealed significant gradients within multiple cohorts. In the 2015 cohort: “wonderful” M = 125.2 versus “not very good” M = 99.0; difference = −26.2 (95% CI: −44.1 to −8.3), *p* = 0.007. In the 2017 cohort: “wonderful” M = 126.1 versus “not very good” M = 105.5; difference = −20.6 (95% CI: −30.5 to −10.7), *p* < 0.001. In the 2016 cohort: “wonderful” M = 125.1 versus “neither good nor bad” M = 111.2; difference = −13.9, *p* = 0.012. Full results: Supplementary Table S18.

EI × Well-being × cohort. Main effect of cohort: *F*(1) = 4.44, *p* = 0.035. Main effect of well-being: *F*(3) = 4.14, *p* = 0.006. Cohort × well-being interaction: *F*(3) = 4.11, *p* = 0.007. The EI gradient across well-being categories was present in multiple cohorts: in the 2015 cohort, “very happy” M = 124.2 versus “unhappy” M = 107.6; difference = −16.5 (95% CI: −26.4 to −6.6), *p* = 0.001; in the 2016 cohort, “very happy” M = 124.2 versus “unhappy” M = 105.1; difference = −19.1 (95% CI: −29.4 to −8.8), *p* < 0.001. Full results: Supplementary Table S20.

EI × Academic stress × cohort. Interaction significant: *F*(1) = 7.27, *p* = 0.007. EI × Satisfaction with medical studies × cohort. Interaction significant: *F*(1) = 11.51, *p* < 0.001. Full ANOVA results: Supplementary Tables S21–S23.

#### 3.2.4 Intrapersonal EI

Inter-cohort differences were borderline significant (*p* = 0.046), with the median stable at Md = 61–62 across all cohorts. The gradient with life evaluation (QOL) was clear: “wonderful” Md = 65 (Q1 = 60, Q3 = 70) versus “not very good” Md = 55 (50, 59); *p* < 0.001. Satisfaction with medical studies showed the strongest gradient: from Md = 49 at satisfaction = 1 to Md = 65 at satisfaction = 10 (p < 0.001). The cohort × QOL interaction for intrapersonal EI was significant (*F*(4) = 6.42, *p* < 0.0001), with gradients evident in the 2014, 2015, and 2017 cohorts.

#### 3.2.5 Interpersonal EI

Clear inter-cohort differences were observed (*p* < 0.001). The highest scores were found in the 2024 (Md = 44, Q1 = 39, Q3 = 48) and 2014 (Md = 43, Q1 = 37, Q3 = 47) cohorts, both significantly higher than the 2015–2018 cohorts (Md = 41). The 2024 cohort differed significantly from all 2015–2018 cohorts (all *p* < 0.0001; Supplementary Table S24).

The gradient with life evaluation was evident: “wonderful” Md = 43 (39, 47) versus “unhappy” Md = 34 (29, 40). The “unhappy” group showed dramatically lower interpersonal EI, differing significantly from the “wonderful” (*p* = 0.002), “successful” (*p* = 0.009), and “quite good” (*p* = 0.034) groups. Academic stress was not associated with interpersonal EI (*p* = 0.047, borderline, no significant post-hoc comparisons).

#### 3.2.6 Comparison of SOC and EI patterns-synthetic summary

SOC showed significant overall associations with adaptation only for satisfaction with medical studies (*p* = 0.029), with significant cohort interactions for three indicators, but post-hoc gradients present exclusively in the 2014 cohort. EI, by contrast, showed strong overall associations with all four indicators (*p* ≤ 0.004), with significant cohort interactions but, in a key contrast with SOC, EI gradients were present across multiple cohorts.

**Figure 3:**
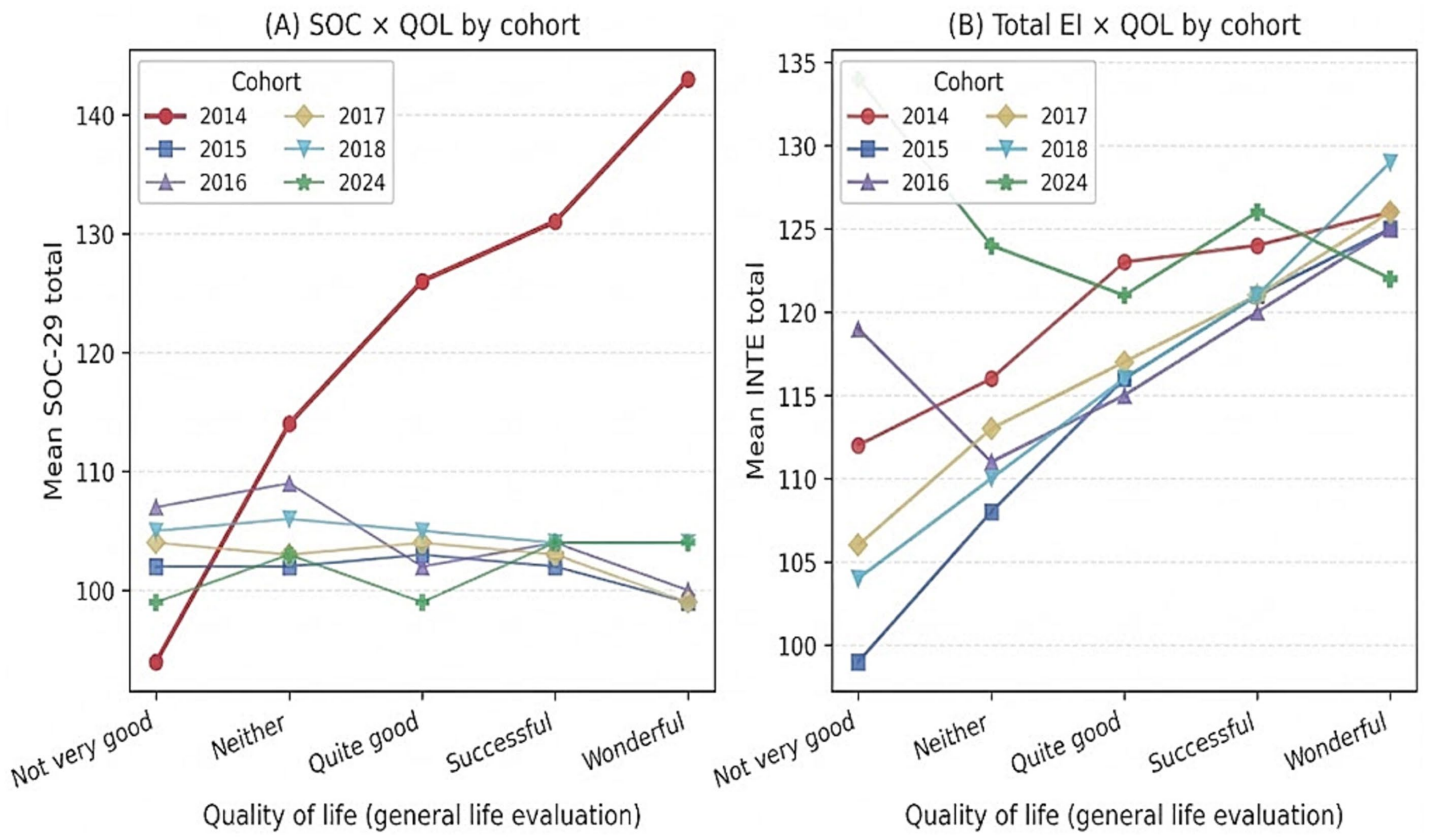
Mean SOC-29 total **(A)** and INTE total **(B)** scores by general life evaluation category, stratified by cohort. Panel A shows the SOC × QOL gradient confined almost exclusively to the 2014 cohort (red); the 2015–2024 cohorts display flat profiles. Panel B shows that EI × QOL gradients are present in multiple cohorts. The asymmetry between panels is the central empirical observation of the study; however, interpretation of panel A is constrained by the markedly lower SOC variance in the 2015–2018 cohorts (see Figure 1 and Discussion).

Hypothesis H4, divergent trends, was confirmed: SOC declined systematically (Md 129 → 101), while total EI increased (Md 118 → 124), and interpersonal EI followed a U-shaped pattern (2014: 43, plateau 2015–2018: 41, increase 2024: 44) (see Tables 2, 3; Figure 3).

**Table 2:** Comparison of SOC and total EI associations with adaptation indicators.

Indicator	SOC: overall *p*	SOC: cohort interaction	EI: overall p	EI: cohort interaction
QOL (life evaluation)	0.090 (n.s.)	*p* = 0.036*	<0.001***	*p* < 0.0001***
Well-being	0.072 (n.s.)	*p* = 0.777 (n.s.)	<0.001***	*p* = 0.007**
Academic stress	0.700 (n.s.)	*p* = 0.037*	0.004**	*p* = 0.007**
Satisfaction with medical studies	0.029*	*p* = 0.050†	<0.001***	*p* < 0.001***

*p < 0.05; **p < 0.01; ***p < 0.001; ^†^borderline (*p* = 0.050); n.s., not significant; SOC, sense of coherence; EI, emotional intelligence (INTE total score); QOL, quality of life. Overall *p* values from Kruskal-Wallis tests. Cohort interaction p values from two-way ANOVA (Type III).

**Table 3:** Cross-cohort trends of SOC and EI across six cohorts of second-year medical students.

Cohort	SOC (Md)	Total EI (Md)	Interpersonal EI (Md)
2014	129	122	43
2015	102	119	41
2016	103	119	41
2017	103	119	41
2018	105	118	41
2024	101	124	44
*Trend*	↓ decline after 2014	↑ increase in 2024	U-shaped (2014 and 2024 highest)

Md, median. SOC, sense of coherence (SOC-29 total score, range 29–203). Total EI and Interpersonal EI from the INTE questionnaire (21). The descriptive labels in the Trend row are intended to summarise the direction of the observed cross-cohort differences; they should not be interpreted as evidence of generational change, which would require measurement invariance testing and longitudinal design.

## 4 Discussion

The main observation of this study is that SOC and EI relate to medical student adaptation in different ways across the six cohorts we examined. In the 2014 cohort, students with higher SOC reported substantially better adaptation. Those rating their lives as “wonderful” scored almost 50 points higher on the SOC-29 than those rating their lives as “not very good.” This pattern did not appear in the 2015–2024 cohorts.

This absence, however, must be interpreted with caution. SOC scores in the later cohorts varied much less than in 2014, and when scores cluster within a narrow range, statistical tests have limited ability to detect any relationship, even one that genuinely exists. From the present data alone, we cannot tell whether the association truly weakened in the more recent cohorts or whether it remains, but is hidden by the reduced variability. Before we can interpret this difference as a change in how SOC supports adaptation across generations, the SOC-29 must first be tested for measurement invariance across cohorts to confirm that it captures the same construct in the same way at each time point. EI showed a different picture. Its association with adaptation was visible in several cohorts, and unlike SOC, EI scores did not lose their variability over time.

The hypotheses were supported to varying degrees.

*H1* was supported for EI (significant associations with all adaptation indicators) but only partially for SOC (significant association only with satisfaction with medical studies).

*H2* (cross-cohort differences in the SOC-adaptation association) was supported descriptively. With the important qualification that variance heterogeneity is an alternative explanation.

*H3* was supported in a relative sense. The EI gradient was present across multiple cohorts while the SOC gradient was restricted to the 2014 cohort.

*H4* (differing cross-cohort trends) was supported descriptively but without permitting causal or generational interpretation, given the cross-cohort design.

These observations raise questions that warrant further investigation. In our earlier longitudinal study conducted on the same university population (13, 14), SOC measured at admission to medical school was found to differentiate styles of career success 4 years after graduation. With the highest-SOC students exhibiting a profile of high quality of life but low career satisfaction and professional competence. The present cross-cohort comparison leaves open whether that differentiating role of SOC was specific to the generation studied in that earlier period (recruited in 1999) or whether it would also be detected in more recent cohorts were the relevant variance preserved. If the cross-cohort difference in SOC-adaptation associations observed here reflects a genuine change, rather than measurement non-invariance, restriction of range, or institution-specific cohort composition effects, then Generalised Resistance Resources (GRRs) may not be fully stable across generations. This interpretation requires future confirmation through measurement invariance testing of the SOC-29 across cohorts, ideally combined with replication at other institutions. We note that such testing is not trivial in the present data: the marked compression of SOC variance in the 2015–2018 cohorts (which is itself one of the central findings) is likely to destabilise multi-group CFA estimates, so invariance testing warrants a dedicated future study rather than a brief check appended to this work. Until such testing is performed, claims about the evolution of GRRs should be treated as hypotheses for future research rather than conclusions of the present study; correspondingly, the cross-cohort SOC differences reported here should be read as descriptive patterns, not as evidence that the underlying construct has changed.

The different patterns observed for SOC and EI may have several explanations, though all remain speculative at this stage. The present study did not directly measure the mechanisms behind these cross-cohort differences, so the following interpretations should be read as candidate hypotheses rather than conclusions.

A first possibility concerns the changing informational environment in which more recent cohorts have grown up. The students entering medical school in 2023 came of age amid information overload, media polarisation, and a series of global crises (23, 24). Such an environment may make it harder to develop a stable, coherent view of the world. This is core of what SOC measures, particularly its comprehensibility component. We note, however, that we did not analyse the SOC-29 subscales separately in this study, so this proposal remains untested in our data. The same generation may, at the same time, have developed stronger emotional skills, partly because seeking psychological help has become more socially accepted and partly because emotional competencies receive more attention in school curricula than they did a decade ago (6, 15). Our data cannot establish a causal link between these social changes and the higher EI scores observed in the 2024 cohort, but the temporal coincidence is compatible with this interpretation.

A second possibility points to medical education itself. Curriculum reforms over the past decade have placed increasing emphasis on communication skills, reflective practice, and patient-centred care. All of which may foster EI. SOC, by contrast, is thought to crystallise during early adulthood through accumulated life experiences rather than through formal teaching (5), and is therefore less likely to respond to changes in the medical curriculum.

A third possibility, which we consider the most important to flag, is that the cross-cohort differences in SOC do not reflect a genuine shift in students’ coherence at all, but rather a measurement issue. SOC scores in the 2015–2018 cohorts varied much less than in 2014, and this compression may stem from selection effects, changes in the recruited population, or other cohort-specific factors. For this reason, conclusions about SOC trends should be drawn cautiously, and greater evidential weight should be placed on the EI findings, where variance remained stable across cohorts.

Taken together, these observations suggest that the resources associated with medical student adaptation may have shifted from the macro level (a global sense that the world is comprehensible and meaningful) toward the micro level (the ability to recognise, regulate, and respond to one’s own and others’ emotions). SOC differentiated adaptation in only one cohort. EI showed a gradient with adaptation in several cohorts and across all four adaptation indicators. This is consistent with recent work pointing to the growing role of emotional competencies in medical education (7, 15), and with our earlier finding that SOC, though predictive in the long term, is not a universal predictor of success but is tied to specific profiles of professional functioning (14).

The practical implications, if these observations are confirmed in future work, are direct. EI is generally considered more malleable than SOC and more amenable to targeted training (6, 15). If it is also more consistently associated with adaptation in contemporary cohorts, then interventions designed to develop emotional competencies may be a more productive use of educational resources than interventions aimed at building a sense of coherence. This aligns with the recent AMEE recommendations (7), which place EI at the centre of medical professional identity formation.

An interesting specific finding is the U-shaped trend in interpersonal EI. The 2014 and 2024 cohorts had the highest scores, while the 2015–2018 cohorts were consistently lower. One speculative interpretation is that high interpersonal EI may arise from different mechanisms across cohorts. Traditional social competencies grounded in face-to-face relationships in the older cohort versus new forms of interpersonal sensitivity shaped in the digital environment in the more recent one. Reversals of the expected direction of the SOC effect were observed in two cohorts. In the 2016 cohort, students with the highest life evaluation had paradoxically lower SOC. In the 2017 cohort, students with the lowest study satisfaction showed higher SOC than those with moderately high satisfaction. These patterns may reflect compensatory idealisation of experiences amid genuinely low sense of coherence, heterogeneity of adaptive mechanisms in more recent cohorts, or artefacts of small subgroup sizes. Regardless of interpretation, these reversals challenge a simple narrative of linear SOC gradient disappearance and suggest a more complex reorganisation of the relationship between SOC and adaptation in more recent cohorts.

An analogous gradient reversal was observed for total EI in the 2024 cohort (Supplementary Table S27). Students rating their life as “not very good” obtained the highest total EI score, higher than those rating it “wonderful.” This finding, based on a small subgroup, requires cautious interpretation, but it suggests that in the most recent cohort, high emotional competence may co-occur with negative life evaluation, apparently contradicting the expected protective pattern of EI and requiring further verification in larger samples.

Because PIF was not directly measured in the present study, we limit our discussion to the constructs we examined. Our findings on EI add to the existing literature linking emotional competencies to outcomes in medical education (7, 15). Whether and how these competencies feed into the longer-term process of PIF is a question for studies that operationalise PIF directly. The practical educational implication of our results is more modest. In cohorts where EI shows broader, more consistent associations with adaptation than SOC does, mentoring programmes may obtain greater leverage by targeting specific emotional competencies than by attempting to build a global sense of meaning.

Beyond the explanations discussed above, the observed divergence between SOC and EI admits several alternative theoretical interpretations that, while speculative, generate testable predictions and may inform future research. These are developed in detail in Appendix 2 and summarised here.

First, a reversed calibration hypothesis proposes that SOC did not lose its protective function but rather lost the inter-individual variance required to demonstrate that function. If contemporary cohorts develop under increasingly homogeneous informational conditions, individual differences in coherence compress, producing null associations regardless of whether SOC retains causal efficacy [cf. restriction of range; (25)].

Second, an algorithmic comprehensibility threshold hypothesis links the abruptness of the SOC decline between 2014 and 2015, followed by a decade-long plateau, to the crossing of critical mass in smartphone and algorithmic social media adoption among adolescents around 2012 (23). That may have selectively undermined the development of the comprehensibility component of SOC while leaving EI, shaped by interpersonal micro-environments, unaffected.

Third, drawing on computational reinforcement learning theory, a cognitive strategy switch hypothesis reconceptualises SOC as a measure of model-based cognitive investment (building a predictive world-model) and EI as the functional analogue of model-free processing (real-time emotional signal detection), suggesting that the generational shift represents an adaptive, population-level switch from model-based to model-free strategies in response to environmental volatility (26, 27).

### 4.1 Limitations

The study has several limitations.

- Our cross-cohort design does not permit causal inference and cannot disentangle cohort, period, age, or selection effects. Observed differences are inevitably confounded with historical events such as the COVID-19 pandemic, the war in Ukraine. Longitudinal tracking of individuals is required to confidently test claims about generational dynamics.
- The lower response rate in the 2024 cohort warrants attention. This decline partly stems from an increased number of available programme places. Nevertheless, it raises the risk of selection bias, as participating students might represent a more motivated or emotionally competent group. Consequently, this provides an alternative explanation for the increased emotional intelligence (EI) observed in 2024 that cannot be ruled out without non-respondent data.
- Reliance on self-report surveys introduces potential common-method and social-desirability biases, despite mitigation efforts like strict anonymity and independent data collection. What is more, adaptation factors were measured using single-item global scales rather than validated multi-item instruments (e.g., WHOQOL). While this preserved comparability across cohorts and minimised respondent burden, it limits the depth of the assessment and precludes the examination of specific well-being subscales.
- A major limitation of the present study is the absence of formal measurement invariance testing for the SOC-29 and INTE instruments across the compared cohorts. Without confirmed invariance, cross-cohort score comparisons cannot rule out differential item functioning or differences in how the underlying constructs were conceptualised by participants in different cohorts. As discussed in section 4, multi-group confirmatory factor analysis on the present data is unlikely to be informative given the marked compression of SOC variance in 2015–2018 (itself a central finding of this study); we therefore identify dedicated invariance testing, with replication at other institutions, as a priority for future work. Accordingly, the cross-cohort comparative findings reported here should be treated as exploratory, providing a preliminary basis for the more rigorous longitudinal and multi-institution studies needed to test these patterns confirmatorily.
- Our large sample sizes mean that some statistically significant between-group differences could in principle lack practical importance. To address this concern, Cohen’s d effect-size estimates were computed for all significant ANOVA post-hoc contrasts and added to Supplementary Tables S3, S6, S9, S18, S20, and S23. Across the 48 significant contrasts, |d| ranged from 0.46 to 2.99 with a median of 1.11, indicating large to very large effects in 38 of 48 cases. The pattern thus does not appear to be driven by a large-N artefact. Additionally, numerous post-hoc tests were conducted. Despite utilising Benjamini-Hochberg corrections, isolated significant results from the ANOVA interaction post-hoc tests, particularly the directional reversals observed in the 2016 and 2017 subgroups, may still reflect Type I error and should not be over-interpreted.

## 5 Conclusion

The landscape of psychological resources supporting medical student adaptation appears to be evolving. In this multi-cohort analysis spanning over a decade, EI emerged as a robust, cross-generational correlate of successful adaptation to medical studies. In contrast, the traditional protective role of SOC was clearly observable only in the earliest cohort. While the apparent disappearance of the SOC gradient in recent cohorts requires further methodological scrutiny, the sustained prominence of EI carries immediate practical implications. As modern medical education increasingly prioritises PIF, our findings suggest that curricula and mentoring programs should pivot from attempting to build a macro-level sense of global coherence toward cultivating actionable, micro-level emotional competencies. Ultimately, equipping the newest generation of physicians to navigate the complexities and crises of contemporary medicine may depend less on static, traditional resilience and more on dynamic emotional plasticity.

## Data Availability

The raw data supporting the conclusions of this article will be made available by the authors, without undue reservation.
