# Supplementary material for: Different resources for different times: sense of coherence and emotional intelligence as correlates of adaptation in six cohorts of medical students
Source: Front Med (Lausanne). 2026 Jun 23;13:1860440. doi: 10.3389/fmed.2026.1860440 (PMC13337364; doi:10.3389/fmed.2026.1860440)
Supplement: Supplementary file 1 [file Table_1.docx]

**Appendix 1**

*Different Resources for Different Times: Sense of Coherence and Emotional Intelligence as Correlates of Adaptation in Six Cohorts of Medical Students*

**Maciej Walkiewicz**

*Department of Psychology, Medical University of Gdańsk, Poland*

**Contents**

This document provides supplementary statistical analyses referenced in the main manuscript. It contains 28 supplementary tables (S1–S28) reporting full descriptive statistics, ANOVA results, and post-hoc comparisons for the cross-cohort analyses of Sense of Coherence (SOC) and Emotional Intelligence (EI) in six cohorts of second-year medical students at the Medical University of Gdańsk, Poland (2014–2024; N = 1,595).

The tables are organised into three sections corresponding to the constructs analysed:

• Section S1 (Tables S1–S10): Sense of Coherence (SOC) analyses.

• Section S2 (Tables S11–S23): Total Emotional Intelligence (INTE total) analyses.

• Section S3 (Tables S24–S28): Intrapersonal and Interpersonal EI and additional analyses.

All analyses were performed in R (version 4.4.1). Between-group comparisons used Kruskal–Wallis tests with Dunn post-hoc tests (Benjamini–Hochberg correction); cohort-by-adaptation interactions were examined with two-way ANOVA (Type III) and Games–Howell post-hoc tests. Full methodological details are provided in Section 2 of the main manuscript. Effect sizes (Cohen's d) for ANOVA post-hoc contrasts have been added in revision to Tables S3, S6, S9, S18, S20, and S23, computed using the simple pooled-SD formula d = (M₁ − M₂)/√((SD₁² + SD₂²)/2) from the descriptive statistics reported in this supplement.

**S1. Sense of Coherence (SOC): descriptive statistics, ANOVA and post-hoc analyses**

**Supplementary Table S1.** SOC descriptive statistics by cohort and quality of life (QOL).

| **QOL** | **2014 (n=297)** | **2015 (n=236)** | **2016 (n=214)** | **2017 (n=284)** | **2018 (n=270)** | **2024 (n=294)** |
| --- | --- | --- | --- | --- | --- | --- |
| Wonderful | 143 (17) | 99 (9) | 100 (9) | 99 (8) | 104 (8) | 104 (17) |
| Successful | 131 (18) | 102 (9) | 104 (10) | 103 (10) | 104 (9) | 104 (16) |
| Quite good | 126 (18) | 103 (6) | 102 (8) | 104 (10) | 105 (10) | 99 (16) |
| Neither | 114 (18) | 102 (13) | 109 (11) | 103 (8) | 106 (11) | 103 (21) |
| Not very good | 94 (16) | 102 (6) | 107 (13) | 104 (8) | 105 (10) | 99 (16) |
| Unhappy | 118 (NA) | 114 (NA) | 111 (11) | 116 (13) | 138 (NA) | 95 (1) |

*Values are M (±SD). QOL = quality of life (general life evaluation). Category “unhappy” (n = 12) was excluded from ANOVA analyses.*

**Supplementary Table S2.** Two-way ANOVA (Type III): SOC × QOL × cohort.

| **Effect** | **Sum of Squares** | **df** | **F** | **p** |
| --- | --- | --- | --- | --- |
| (Intercept) | 6,627.645 | 1 | 26.92 | < 0.0001 |
| Cohort | 6,017.129 | 1 | 24.44 | < 0.0001 |
| QOL | 2,542.950 | 4 | 2.58 | 0.036 |
| Cohort × QOL | 2,535.984 | 4 | 2.58 | 0.036 |

*Type III sums of squares. SOC = sense of coherence; QOL = quality of life.*

**Supplementary Table S3.** Post-hoc Games-Howell tests: SOC × QOL — significant results.

| **Cohort** | **Group 1** | **M (SD)** | **Group 2** | **M (SD)** | **Diff (95% CI)** | **p** | **d** |
| --- | --- | --- | --- | --- | --- | --- | --- |
| 2014 | Successful | 130.7 (17.6) | Wonderful | 142.8 (17.0) | −12.1 (−20.7; −3.5) | 0.001 | -0.70 |
| 2014 | Quite good | 126.0 (17.7) | Wonderful | 142.8 (17.0) | −16.8 (−26.4; −7.2) | < 0.001 | -0.97 |
| 2014 | Neither | 114.2 (18.4) | Wonderful | 142.8 (17.0) | −28.7 (−42.5; −14.8) | < 0.001 | -1.61 |
| 2014 | Not very good | 94.0 (15.6) | Wonderful | 142.8 (17.0) | −48.8 (−71.6; −26.0) | < 0.001 | -2.99 |
| 2014 | Neither | 114.2 (18.4) | Successful | 130.7 (17.6) | −16.6 (−29.0; −4.1) | 0.003 | -0.92 |
| 2014 | Not very good | 94.0 (15.6) | Successful | 130.7 (17.6) | −36.7 (−58.7; −14.8) | < 0.001 | -2.21 |
| 2014 | Not very good | 94.0 (15.6) | Quite good | 126.0 (17.7) | −32.0 (−54.4; −9.6) | 0.001 | -1.92 |
| 2016 | Neither | 109.1 (10.6) | Wonderful | 100.0 (9.4) | 9.1 (1.4; 16.8) | 0.012 | +0.91 |

*Diff = mean difference (95% confidence interval). Only statistically significant comparisons shown.*

**Supplementary Table S4.** SOC descriptive statistics by cohort and academic stress.

| **Stress** | **2014** | **2015** | **2016** | **2017** | **2018** | **2024** |
| --- | --- | --- | --- | --- | --- | --- |
| 2 | 148 (16) | 107 (4) | 97 (1) | 99 (3) | 106 (8) | 85 (21) |
| 3 | 151 (16) | 102 (13) | 101 (11) | 104 (8) | 106 (7) | 104 (16) |
| 4 | 138 (17) | 96 (9) | 102 (8) | 105 (9) | 104 (7) | 100 (22) |
| 5 | 139 (17) | 102 (9) | 102 (11) | 100 (10) | 104 (10) | 104 (18) |
| 6 | 131 (17) | 103 (9) | 103 (11) | 102 (9) | 106 (9) | 103 (15) |
| 7 | 133 (19) | 103 (7) | 106 (10) | 103 (9) | 105 (9) | 100 (18) |
| 8 | 126 (18) | 103 (10) | 103 (9) | 104 (10) | 103 (10) | 102 (15) |
| 9 | 121 (17) | 102 (8) | 103 (10) | 102 (10) | 104 (12) | 105 (15) |

*Values are M (±SD). Academic stress rated 1–10 (VAS). Category 1 (n = 4) excluded from ANOVA.*

**Supplementary Table S5.** Two-way ANOVA (Type III): SOC × academic stress × cohort.

| **Effect** | **Sum of Squares** | **df** | **F** | **p** |
| --- | --- | --- | --- | --- |
| (Intercept) | 31,072.870 | 1 | 125.96 | < 0.0001 |
| Cohort | 29,427.114 | 1 | 119.28 | < 0.0001 |
| Academic stress | 3,713.422 | 7 | 2.15 | 0.036 |
| Cohort × Stress | 3,702.072 | 7 | 2.14 | 0.037 |

*Type III sums of squares.*

**Supplementary Table S6.** Post-hoc Games-Howell: SOC × academic stress — cohort 2014.

| **Stress 1** | **M (SD)** | **Stress 2** | **M (SD)** | **Diff (95% CI)** | **p** | **d** |
| --- | --- | --- | --- | --- | --- | --- |
| 6 | 131.5 (16.6) | 3 | 150.9 (16.2) | −19.4 (−37.7; −1.2) | 0.029 | -1.18 |
| 7 | 133.1 (19.3) | 3 | 150.9 (16.2) | −17.9 (−34.8; −0.9) | 0.031 | -1.00 |
| 8 | 126.1 (18.0) | 3 | 150.9 (16.2) | −24.9 (−41.2; −8.5) | < 0.001 | -1.45 |
| 9 | 121.1 (17.4) | 3 | 150.9 (16.2) | −29.8 (−46.3; −13.3) | < 0.0001 | -1.77 |
| 8 | 126.1 (18.0) | 5 | 139.2 (17.1) | −13.2 (−24.9; −1.4) | 0.017 | -0.75 |
| 9 | 121.1 (17.4) | 5 | 139.2 (17.1) | −18.1 (−30.0; −6.1) | < 0.001 | -1.05 |
| 9 | 121.1 (17.4) | 7 | 133.1 (19.3) | −11.9 (−23.4; −0.4) | 0.036 | -0.65 |

*Diff = mean difference (95% CI). Only significant comparisons from cohort 2014 shown.*

**Supplementary Table S7.** SOC descriptive statistics by cohort and satisfaction with medical studies.

| **Satisfaction** | **2014** | **2015** | **2016** | **2017** | **2018** | **2024** |
| --- | --- | --- | --- | --- | --- | --- |
| 2 | 92 (NA) | 96 (11) | 99 (6) | 106 (12) | 108 (9) | 107 (12) |
| 3 | 120 (NA) | 100 (8) | 101 (4) | 106 (7) | 104 (13) | 96 (15) |
| 4 | 122 (22) | 101 (10) | 101 (10) | 103 (7) | 103 (11) | 94 (27) |
| 5 | 122 (11) | 101 (8) | 106 (12) | 105 (9) | 106 (11) | 101 (13) |
| 6 | 121 (21) | 105 (12) | 107 (9) | 104 (11) | 103 (9) | 104 (18) |
| 7 | 127 (18) | 100 (7) | 101 (9) | 102 (10) | 104 (10) | 103 (17) |
| 8 | 131 (20) | 103 (8) | 105 (8) | 100 (9) | 105 (9) | 101 (17) |
| 9 | 136 (16) | 103 (8) | 100 (10) | 103 (9) | 104 (9) | 102 (16) |

*Values are M (±SD). Satisfaction rated 1–10. Category 1 (n = 15) excluded from ANOVA.*

**Supplementary Table S8.** Two-way ANOVA (Type III): SOC × satisfaction with medical studies × cohort.

| **Effect** | **Sum of Squares** | **df** | **F** | **p** |
| --- | --- | --- | --- | --- |
| (Intercept) | 8,159.382 | 1 | 35.85 | < 0.0001 |
| Cohort | 7,429.169 | 1 | 32.64 | < 0.0001 |
| Satisfaction | 3,228.077 | 7 | 2.03 | 0.049 |
| Cohort × Satisfaction | 3,219.780 | 7 | 2.02 | 0.050 |

*Type III sums of squares.*

**Supplementary Table S9.** Post-hoc Games-Howell: SOC × satisfaction — significant results.

| **Cohort** | **Group 1** | **M (SD)** | **Group 2** | **M (SD)** | **Diff (95% CI)** | **p** | **d** |
| --- | --- | --- | --- | --- | --- | --- | --- |
| 2014 | Sat = 9 | 135.8 (15.8) | Sat = 6 | 121.2 (20.8) | 14.6 (0.3; 28.9) | 0.042 | +0.79 |
| 2017 | Sat = 7 | 101.5 (10.3) | Sat = 1 | 116.0 (9.0) | −14.5 (−27.1; −1.8) | 0.012 | -1.50 |
| 2017 | Sat = 8 | 100.5 (8.8) | Sat = 1 | 116.0 (9.0) | −15.5 (−28.1; −2.9) | 0.005 | -1.74 |

*Sat = satisfaction with medical studies. Diff = mean difference (95% CI).*

**Supplementary Table S10.** Two-way ANOVA (Type III): SOC × well-being × cohort.

| **Effect** | **Sum of Squares** | **df** | **F** | **p** |
| --- | --- | --- | --- | --- |
| (Intercept) | 17,935.079 | 1 | 72.60 | < 0.0001 |
| Cohort | 16,649.854 | 1 | 67.40 | < 0.0001 |
| Well-being | 274.288 | 3 | 0.37 | 0.775 |
| Cohort × Well-being | 271.716 | 3 | 0.37 | 0.777 |

*Interaction not significant — no post-hoc tests performed.*

**S2. Total Emotional Intelligence (INTE total): descriptive statistics, ANOVA and post-hoc analyses**

**Supplementary Table S11.** Total EI descriptive statistics by cohort: Md (Q1, Q3).

| **Cohort** | **n** | **Md** | **Q1** | **Q3** |
| --- | --- | --- | --- | --- |
| 2014 | 297 | 122 | 110 | 131 |
| 2015 | 236 | 119 | 112 | 126 |
| 2016 | 205 | 119 | 111 | 126 |
| 2017 | 284 | 119 | 111 | 126 |
| 2018 | 270 | 118 | 110 | 126 |
| 2024 | 262 | 124 | 115 | 132 |

*EI = emotional intelligence (INTE total score). Md = median; Q1, Q3 = first and third quartile.*

**Supplementary Table S12.** Post-hoc Dunn tests (Benjamini-Hochberg): total EI between cohorts.

| **Cohort 1** | **Mean rank** | **Cohort 2** | **Mean rank** | **Statistic** | **p adj.** |
| --- | --- | --- | --- | --- | --- |
| 2015 | 744.72 | 2024 | 900.74 | 3.88 | 0.001 |
| 2016 | 724.77 | 2024 | 900.74 | 4.21 | < 0.001 |
| 2017 | 743.87 | 2024 | 900.74 | 4.08 | < 0.001 |
| 2018 | 718.20 | 2024 | 900.74 | 4.69 | < 0.0001 |

*Only significant pairwise comparisons shown. p adj. = Benjamini-Hochberg adjusted p-value.*

**Supplementary Table S13.** Post-hoc Dunn tests (Benjamini-Hochberg): total EI × QOL.

| **Group 1** | **n** | **Rank** | **Group 2** | **n** | **Rank** | **Stat.** | **p adj.** |
| --- | --- | --- | --- | --- | --- | --- | --- |
| Wonderful | 208 | 946 | Successful | 694 | 823 | −3.60 | 0.002 |
| Wonderful | 208 | 946 | Quite good | 423 | 666 | −7.61 | < 0.0001 |
| Wonderful | 208 | 946 | Neither | 130 | 524 | −8.69 | < 0.0001 |
| Wonderful | 208 | 946 | Not very good | 40 | 359 | −7.83 | < 0.0001 |
| Wonderful | 208 | 946 | Unhappy | 12 | 355 | −4.58 | < 0.0001 |
| Successful | 694 | 823 | Quite good | 423 | 666 | −5.82 | < 0.0001 |
| Successful | 694 | 823 | Neither | 130 | 524 | −7.18 | < 0.0001 |
| Successful | 694 | 823 | Not very good | 40 | 359 | −6.56 | < 0.0001 |
| Successful | 694 | 823 | Unhappy | 12 | 355 | −3.69 | 0.002 |
| Quite good | 423 | 666 | Neither | 130 | 524 | −3.26 | 0.006 |
| Quite good | 423 | 666 | Not very good | 40 | 359 | −4.27 | < 0.001 |

*Only significant comparisons shown. QOL = quality of life (general life evaluation).*

**Supplementary Table S14.** Post-hoc Dunn tests (Benjamini-Hochberg): total EI × well-being.

| **Group 1** | **Mean rank** | **Group 2** | **Mean rank** | **Statistic** | **p adj.** |
| --- | --- | --- | --- | --- | --- |
| Very happy | 909 | Quite happy | 791 | −3.74 | < 0.001 |
| Very happy | 909 | Not very happy | 624 | −8.04 | < 0.0001 |
| Very happy | 909 | Unhappy | 521 | −7.27 | < 0.0001 |
| Quite happy | 791 | Not very happy | 624 | −6.15 | < 0.0001 |
| Quite happy | 791 | Unhappy | 521 | −5.59 | < 0.0001 |
| Not very happy | 624 | Unhappy | 521 | −2.03 | 0.043 |

*All pairwise comparisons were statistically significant, confirming a monotonic four-level gradient.*

**Supplementary Table S15.** Post-hoc Dunn tests (Benjamini-Hochberg): total EI × academic stress.

| **Stress group 1** | **Mean rank** | **Stress group 2** | **Mean rank** | **Statistic** | **p adj.** |
| --- | --- | --- | --- | --- | --- |
| 3 | 903 | 8 | 686 | −4.08 | 0.002 |
| 3 | 903 | 9 | 714 | −3.48 | 0.022 |

*Only significant comparisons shown. Remaining pairwise comparisons were not significant.*

**Supplementary Table S16.** Post-hoc Dunn tests (Benjamini-Hochberg): total EI × satisfaction with medical studies — all significant comparisons.

| **Sat 1** | **n** | **Rank** | **Sat 2** | **n** | **Rank** | **Stat.** | **p adj.** |
| --- | --- | --- | --- | --- | --- | --- | --- |
| 1 | 15 | 361 | 7 | 324 | 732 | 3.22 | 0.035 |
| 1 | 15 | 361 | 8 | 368 | 778 | 3.63 | 0.009 |
| 1 | 15 | 361 | 9 | 157 | 878 | 4.39 | < 0.001 |
| 1 | 15 | 361 | 10 | 198 | 936 | 4.92 | < 0.0001 |
| 2 | 36 | 458 | 9 | 157 | 878 | 3.97 | 0.003 |
| 2 | 36 | 458 | 10 | 198 | 936 | 4.79 | < 0.001 |
| 3 | 60 | 539 | 7 | 324 | 732 | 3.15 | 0.043 |
| 3 | 60 | 539 | 8 | 368 | 778 | 3.93 | 0.003 |
| 3 | 60 | 539 | 9 | 157 | 878 | 5.12 | < 0.0001 |
| 3 | 60 | 539 | 10 | 198 | 936 | 6.18 | < 0.0001 |
| 4 | 69 | 583 | 8 | 368 | 778 | 3.41 | 0.018 |
| 4 | 69 | 583 | 9 | 157 | 878 | 4.69 | < 0.001 |
| 4 | 69 | 583 | 10 | 198 | 936 | 5.80 | < 0.0001 |
| 5 | 121 | 697 | 9 | 157 | 878 | 3.43 | 0.018 |
| 5 | 121 | 697 | 10 | 198 | 936 | 4.75 | < 0.001 |
| 6 | 161 | 686 | 9 | 157 | 878 | 3.92 | 0.003 |
| 6 | 161 | 686 | 10 | 198 | 936 | 5.40 | < 0.0001 |
| 7 | 324 | 732 | 9 | 157 | 878 | 3.44 | 0.017 |
| 7 | 324 | 732 | 10 | 198 | 936 | 5.19 | < 0.0001 |
| 8 | 368 | 778 | 10 | 198 | 936 | 4.13 | 0.001 |

*Sat = satisfaction with medical studies (1–10). Only significant comparisons shown.*

**Supplementary Table S17.** Two-way ANOVA (Type III): total EI × QOL × cohort.

| **Effect** | **Sum of Squares** | **df** | **F** | **p** |
| --- | --- | --- | --- | --- |
| (Intercept) | 2,038.065 | 1 | 14.33 | < 0.001 |
| Cohort | 2,412.025 | 1 | 16.96 | < 0.0001 |
| QOL | 3,618.328 | 4 | 6.36 | < 0.0001 |
| Cohort × QOL | 3,596.383 | 4 | 6.32 | < 0.0001 |

*Category “unhappy” (n = 12) excluded from analysis.*

**Supplementary Table S18.** Post-hoc Games-Howell: total EI × QOL × cohort — all significant results.

| **Cohort** | **Group 1** | **M (SD)** | **Group 2** | **M (SD)** | **Diff (95% CI)** | **p** | **d** |
| --- | --- | --- | --- | --- | --- | --- | --- |
| 2015 | Quite good | 115.7 (10.4) | Wonderful | 125.2 (10.5) | −9.5 (−16.4; −2.7) | 0.002 | -0.91 |
| 2015 | Neither | 107.8 (15.2) | Wonderful | 125.2 (10.5) | −17.4 (−25.6; −9.1) | < 0.001 | -1.33 |
| 2015 | Not v. good | 99.0 (9.5) | Wonderful | 125.2 (10.5) | −26.2 (−44.1; −8.3) | 0.007 | -2.62 |
| 2015 | Quite good | 115.7 (10.4) | Successful | 121.5 (9.8) | −5.8 (−10.5; −1.1) | 0.007 | -0.57 |
| 2015 | Neither | 107.8 (15.2) | Successful | 121.5 (9.8) | −13.6 (−20.2; −7.1) | < 0.001 | -1.07 |
| 2015 | Not v. good | 99.0 (9.5) | Successful | 121.5 (9.8) | −22.5 (−39.6; −5.3) | 0.036 | -2.33 |
| 2015 | Neither | 107.8 (15.2) | Quite good | 115.7 (10.4) | −7.9 (−15.0; −0.7) | 0.023 | -0.61 |
| 2016 | Quite good | 114.6 (9.8) | Wonderful | 125.1 (10.4) | −10.5 (−17.9; −3.2) | 0.011 | -1.04 |
| 2016 | Neither | 111.2 (10.6) | Wonderful | 125.1 (10.4) | −13.9 (−23.7; −4.1) | 0.012 | -1.32 |
| 2017 | Quite good | 117.0 (10.9) | Wonderful | 126.1 (11.4) | −9.1 (−15.1; −3.1) | 0.004 | -0.82 |
| 2017 | Neither | 113.1 (11.4) | Wonderful | 126.1 (11.4) | −13.0 (−21.0; −5.0) | 0.001 | -1.14 |
| 2017 | Not v. good | 105.5 (10.1) | Wonderful | 126.1 (11.4) | −20.6 (−30.5; −10.7) | < 0.001 | -1.91 |
| 2017 | Neither | 113.1 (11.4) | Successful | 120.8 (10.2) | −7.8 (−14.4; −1.1) | 0.013 | -0.71 |
| 2017 | Not v. good | 105.5 (10.1) | Successful | 120.8 (10.2) | −15.3 (−24.2; −6.5) | < 0.001 | -1.51 |

*Diff = mean difference (95% CI). Not v. good = not very good.*

**Supplementary Table S19.** Two-way ANOVA (Type III): total EI × well-being × cohort.

| **Effect** | **Sum of Squares** | **df** | **F** | **p** |
| --- | --- | --- | --- | --- |
| (Intercept) | 431.411 | 1 | 2.86 | 0.091 |
| Cohort | 669.369 | 1 | 4.44 | 0.035 |
| Well-being | 1,870.605 | 3 | 4.14 | 0.006 |
| Cohort × Well-being | 1,858.694 | 3 | 4.11 | 0.007 |

*Type III sums of squares.*

**Supplementary Table S20.** Post-hoc Games-Howell: total EI × well-being × cohort — significant results.

| **Cohort** | **Group 1** | **M (SD)** | **Group 2** | **M (SD)** | **Diff (95% CI)** | **p** | **d** |
| --- | --- | --- | --- | --- | --- | --- | --- |
| 2014 | Not v. happy | 119.6 (12.8) | Very happy | 127.7 (17.2) | −8.1 (−14.7; −1.5) | 0.010 | -0.53 |
| 2015 | Not v. happy | 114.4 (11.8) | Very happy | 124.2 (9.4) | −9.8 (−16.0; −3.6) | 0.003 | -0.92 |
| 2015 | Unhappy | 107.6 (14.9) | Very happy | 124.2 (9.4) | −16.5 (−26.4; −6.6) | 0.001 | -1.33 |
| 2015 | Not v. happy | 114.4 (11.8) | Q. happy | 119.7 (11.3) | −5.3 (−10.3; −0.4) | 0.027 | -0.46 |
| 2015 | Unhappy | 107.6 (14.9) | Q. happy | 119.7 (11.3) | −12.1 (−21.2; −2.9) | 0.043 | -0.92 |
| 2016 | Not v. happy | 115.6 (12.7) | Very happy | 124.2 (10.2) | −8.6 (−15.9; −1.3) | 0.013 | -0.75 |
| 2016 | Unhappy | 105.1 (17.8) | Very happy | 124.2 (10.2) | −19.1 (−29.4; −8.8) | < 0.001 | -1.32 |
| 2016 | Unhappy | 105.1 (17.8) | Q. happy | 119.5 (12.0) | −14.3 (−24.1; −4.5) | 0.010 | -0.95 |

*Not v. happy = not very happy. Q. happy = quite happy. Diff = mean difference (95% CI).*

**Supplementary Table S21.** Two-way ANOVA (Type III): total EI × academic stress × cohort.

| **Effect** | **Sum of Squares** | **df** | **F** | **p** |
| --- | --- | --- | --- | --- |
| (Intercept) | 775.052 | 1 | 4.84 | 0.028 |
| Cohort | 648.253 | 1 | 4.05 | 0.044 |
| Academic stress | 1,168.735 | 1 | 7.30 | 0.007 |
| Cohort × Stress | 1,164.042 | 1 | 7.27 | 0.007 |

*Type III sums of squares.*

**Supplementary Table S22.** Two-way ANOVA (Type III): total EI × satisfaction with medical studies × cohort.

| **Effect** | **Sum of Squares** | **df** | **F** | **p** |
| --- | --- | --- | --- | --- |
| (Intercept) | 1,986.342 | 1 | 13.48 | < 0.001 |
| Cohort | 2,136.913 | 1 | 14.50 | < 0.001 |
| Satisfaction | 1,710.801 | 1 | 11.61 | < 0.001 |
| Cohort × Satisfaction | 1,696.995 | 1 | 11.51 | < 0.001 |

*Type III sums of squares.*

**Supplementary Table S23.** Post-hoc Games-Howell: total EI × satisfaction × cohort — significant results.

| **Cohort** | **Group 1** | **M (SD)** | **Group 2** | **M (SD)** | **Diff (95% CI)** | **p** | **d** |
| --- | --- | --- | --- | --- | --- | --- | --- |
| 2014 | Sat=9 | 125.8 (11.9) | Sat=6 | 114.3 (12.2) | 11.5 (1.0; 21.9) | 0.021 | +0.95 |
| 2014 | Sat=10 | 127.8 (15.6) | Sat=6 | 114.3 (12.2) | 13.5 (3.7; 23.3) | 0.011 | +0.96 |
| 2015 | Sat=10 | 124.9 (12.8) | Sat=2 | 107.5 (15.9) | 17.4 (1.6; 33.2) | 0.020 | +1.21 |
| 2015 | Sat=9 | 123.2 (6.1) | Sat=3 | 108.1 (12.4) | 15.1 (1.7; 28.4) | 0.014 | +1.55 |
| 2015 | Sat=10 | 124.9 (12.8) | Sat=3 | 108.1 (12.4) | 16.8 (4.3; 29.3) | 0.011 | +1.33 |
| 2017 | Sat=5 | 116.7 (12.7) | Sat=1 | 100.3 (12.5) | 16.4 (0.8; 31.9) | 0.030 | +1.30 |
| 2017 | Sat=6 | 119.5 (11.1) | Sat=1 | 100.3 (12.5) | 19.2 (3.6; 34.7) | 0.043 | +1.62 |
| 2017 | Sat=8 | 121.6 (10.7) | Sat=1 | 100.3 (12.5) | 21.3 (6.4; 36.2) | 0.003 | +1.83 |

*Sat = satisfaction with medical studies. Diff = mean difference (95% CI).*

**S3. Intrapersonal and Interpersonal Emotional Intelligence, and additional analyses**

**Supplementary Table S24.** Post-hoc Dunn tests (Benjamini-Hochberg): interpersonal EI between cohorts.

| **Cohort 1** | **Mean rank** | **Cohort 2** | **Mean rank** | **Statistic** | **p adj.** |
| --- | --- | --- | --- | --- | --- |
| 2014 | 829 | 2015 | 703 | −3.24 | 0.012 |
| 2014 | 829 | 2016 | 718 | −2.74 | 0.049 |
| 2014 | 829 | 2018 | 695 | −3.55 | 0.004 |
| 2014 | 829 | 2024 | 947 | 3.11 | 0.017 |
| 2015 | 703 | 2024 | 947 | 6.09 | < 0.0001 |
| 2016 | 718 | 2024 | 947 | 5.50 | < 0.0001 |
| 2017 | 751 | 2024 | 947 | 5.12 | < 0.0001 |
| 2018 | 695 | 2024 | 947 | 6.49 | < 0.0001 |

*p adj. = Benjamini-Hochberg adjusted p-value.*

**Supplementary Table S25.** Intrapersonal EI descriptive statistics by adaptation indicators: Md (Q1, Q3).

| **Indicator** | **Category** | **Md** | **Q1** | **Q3** | **p (KW)** |
| --- | --- | --- | --- | --- | --- |
| QOL | Wonderful | 65 | 60 | 70 | < 0.001 |
| QOL | Successful | 63 | 59 | 67 |  |
| QOL | Quite good | 60 | 56 | 64 |  |
| QOL | Neither | 58 | 52 | 64 |  |
| QOL | Not very good | 55 | 50 | 59 |  |
| QOL | Unhappy | 55 | 47 | 64 |  |
| Well-being | Very happy | 65 | 60 | 69 | < 0.001 |
| Well-being | Quite happy | 62 | 58 | 67 |  |
| Well-being | Not v. happy | 60 | 55 | 65 |  |
| Well-being | Unhappy | 57 | 51 | 63 |  |
| Stress | (overall) | — | — | — | 0.002 |
| Satisfaction | 1 | 49 | 44 | 61 | < 0.001 |
| Satisfaction | 5 | 61 | 56 | 65 |  |
| Satisfaction | 9 | 64 | 60 | 68 |  |
| Satisfaction | 10 | 65 | 61 | 70 |  |

*KW = Kruskal-Wallis test. Md = median. Satisfaction = satisfaction with medical studies (1–10).*

**Supplementary Table S26.** Interpersonal EI descriptive statistics by adaptation indicators: Md (Q1, Q3).

| **Indicator** | **Category** | **Md** | **Q1** | **Q3** | **p (KW)** |
| --- | --- | --- | --- | --- | --- |
| QOL | Wonderful | 43 | 39 | 47 | < 0.001 |
| QOL | Successful | 42 | 39 | 46 |  |
| QOL | Quite good | 41 | 38 | 45 |  |
| QOL | Neither | 40 | 37 | 44 |  |
| QOL | Not very good | 40 | 36 | 44 |  |
| QOL | Unhappy | 34 | 29 | 40 |  |
| Well-being | Very happy | 43 | 40 | 47 | < 0.001 |
| Well-being | Quite happy | 42 | 39 | 45 |  |
| Well-being | Not v. happy | 41 | 38 | 44 |  |
| Well-being | Unhappy | 40 | 36 | 46 |  |
| Stress | (overall) | — | — | — | 0.047 |
| Satisfaction | 1 | 38 | 33 | 40 | < 0.001 |
| Satisfaction | 5 | 41 | 38 | 45 |  |
| Satisfaction | 9 | 43 | 40 | 47 |  |
| Satisfaction | 10 | 43 | 40 | 47 |  |

*KW = Kruskal-Wallis test. Md = median. Satisfaction = satisfaction with medical studies (1–10).*

**Supplementary Table S27.** Total EI descriptive statistics by cohort and QOL: M (±SD).

| **QOL** | **2014** | **2015** | **2016** | **2017** | **2018** | **2024** |
| --- | --- | --- | --- | --- | --- | --- |
| Wonderful | 126 (17) | 125 (11) | 125 (10) | 126 (11) | 129 (8) | 122 (14) |
| Successful | 124 (12) | 121 (10) | 120 (13) | 121 (10) | 121 (10) | 126 (12) |
| Quite good | 123 (12) | 116 (10) | 115 (10) | 117 (11) | 116 (11) | 121 (15) |
| Neither | 116 (12) | 108 (15) | 111 (11) | 113 (11) | 110 (12) | 124 (12) |
| Not very good | 112 (12) | 99 (10) | 119 (1) | 106 (10) | 104 (7) | 134 (12) |
| Unhappy | 116 (NA) | 106 (NA) | 94 (23) | 105 (16) | 129 (NA) | 105 (19) |

*Values are M (±SD). QOL = quality of life (general life evaluation).*

**Supplementary Table S28.** Total EI descriptive statistics by cohort and satisfaction with medical studies: M (±SD).

| **Satisfaction** | **2014** | **2015** | **2016** | **2017** | **2018** | **2024** |
| --- | --- | --- | --- | --- | --- | --- |
| 1 | 89 (NA) | NA | 105 (33) | 100 (13) | 98 (NA) | 127 (12) |
| 2 | 122 (NA) | 108 (16) | 115 (15) | 115 (13) | 115 (17) | 120 (11) |
| 3 | 98 (NA) | 108 (12) | 106 (10) | 116 (9) | 116 (11) | 127 (11) |
| 4 | 122 (13) | 115 (9) | 115 (14) | 114 (11) | 114 (12) | 121 (16) |
| 5 | 124 (9) | 119 (9) | 112 (21) | 117 (13) | 114 (12) | 121 (14) |
| 6 | 114 (12) | 117 (15) | 118 (10) | 119 (11) | 116 (12) | 127 (15) |
| 7 | 123 (13) | 118 (11) | 117 (11) | 118 (10) | 118 (9) | 122 (14) |
| 8 | 123 (12) | 119 (10) | 120 (11) | 122 (11) | 118 (12) | 123 (13) |
| 9 | 126 (12) | 123 (6) | 123 (10) | 121 (10) | 121 (12) | 126 (12) |
| 10 | 128 (16) | 125 (13) | 121 (12) | 126 (13) | 127 (10) | 123 (16) |

*Values are M (±SD). Satisfaction = satisfaction with medical studies (1–10). NA = insufficient data for SD calculation.*
