# Supplementary material for: Different resources for different times: sense of coherence and emotional intelligence as correlates of adaptation in six cohorts of medical students
Source: Front Med (Lausanne). 2026 Jun 23;13:1860440. doi: 10.3389/fmed.2026.1860440 (PMC13337364; doi:10.3389/fmed.2026.1860440)
Supplement: Supplementary file 2 [file Table_2.DOCX]

**Appendix 2**

Alternative Theoretical Frameworks for the Observed Cross-Cohort Differences in Sense of Coherence and Emotional Intelligence

*Supplementary theoretical material for:*

*"Different Resources for Different Times: Sense of Coherence and Emotional Intelligence as Correlates of Adaptation in Six Cohorts of Medical Students*

**Scope and status of this appendix**

The main text offers several candidate explanations grounded in educational and socio-cultural change, while maintaining explicit epistemic caution about causal and generational interpretations. The present appendix develops three additional theoretical frameworks that draw on psychometrics, digital ecology, and computational neuroscience. These frameworks are deliberately speculative. They do not constitute claims derived from the present data. Readers should treat this material as a set of hypotheses for future research rather than conclusions of the present study.

**1. The Reversed Calibration Hypothesis**

SOC scores in the 2014 cohort varied widely (SD ≈ 16–19), whereas in the 2015–2018 cohorts they clustered within a narrow range (SD ≈ 7–11). The standard explanation treats this as a methodological artefact. The reversed calibration hypothesis offers a different reading. The variance compression is itself the phenomenon may be explained.

The argument rests on how SOC develops. According to Antonovsky (1), SOC crystallises during late adolescence through accumulated life experiences that shape whether one perceives the world as comprehensible, manageable, and meaningful. The 2014 cohort (formative years approximately 2008–2012) grew up in informationally diverse conditions. Some adolescents encountered structured, consistent environments, others did not. This diversity produced wide individual differences in SOC. Students with the highest scores, those who had built the most robust models of their world, showed the clearest adaptive benefit.

We propose that for subsequent cohorts the formative environment became uniform in a specific way. Not impoverished, but uniformly unpredictable. Algorithmic content delivery, social media, and rapid news cycles exposed all adolescents to structurally similar patterns of informational inconsistency, regardless of individual circumstances. When everyone receives the same kind of chaotic input, individual differences in perceived comprehensibility compress. Not because the questionnaire fails, but because the environmental variation that normally produces different SOC levels has itself disappeared.

This compression has a direct statistical consequence. When scores on a predictor cluster within a narrow range, correlations with any outcome attenuate toward zero, even if the underlying causal relationship is intact (5, 6). SOC may therefore still matter for adaptation but simply lack the variance needed to show it.

EI develops differently. Its primary inputs: family dynamics, peer relationships, intimate social interactions, remain heterogeneous even when the macro-informational environment has become uniform. This is why EI retains enough individual variation to predict adaptation across all cohorts.

**2. The Algorithmic Comprehensibility Threshold Hypothesis**

The first hypothesis explains why variance compressed. This hypothesis explains when and why the compression occurred with such abruptness. The transition between the 2014 and 2015 cohorts is not gradual, it is a step function. Median SOC dropped from 129 to 102 (a 27-point decline), and then remained essentially flat (101–105) for a full decade. This pattern is consistent with a threshold effect. Something changed in the formative environment of these two adjacent cohorts that fundamentally altered the conditions for SOC development.

We propose that this threshold corresponds to the mass adoption of smartphones and algorithmic social media among Polish adolescents around 2012. The 2014 cohort entered university in September 2012. Their formative years for SOC crystallisation (approximately ages 15–18) fell in 2008–2012, a period that preceded or coincided with the very earliest stages of smartphone saturation. The 2015 cohort entered in September 2013, with formative years approximately 2009–2013, straddling the inflection point. This chronology aligns precisely with what Twenge (7) has described as the global "smartphone inflection point". The moment when smartphone ownership among adolescents exceeded 50% and social media use became a near-universal feature of daily life. In Poland specifically, smartphone penetration among the general population exceeded 50% around 2014–2015, but among adolescents and young adults (16–24 years) the threshold was crossed earlier, around 2012–2013, coinciding with the entry of affordable Android devices into the mass market (8, 9). Eurostat data confirm that daily internet use among young people in Poland overtook daily computer use in 2012, a pattern consistent with the shift to mobile-first information consumption (10). Facebook surpassed 10 million Polish users around 2011–2012, with the highest penetration in the 16–24 age group (9).

The theoretical link to SOC operates through the comprehensibility component. Antonovsky (1) defined comprehensibility as the expectation that environmental stimuli are "ordered, consistent, structured, and clear" rather than "chaotic, disordered, random, accidental, and inexplicable." Pre-algorithmic information environments, television, newspapers, textbooks, face-to-face conversation, were biased and incomplete, but they were internally consistent. A teenager in 2010 could construct a coherent, if imperfect, model of the world from a stable set of sources. Different teenagers built different models (some stronger, some weaker), producing high inter-individual variance in comprehensibility and, by extension, in total SOC.

Algorithmic information environments fundamentally alter this ecology. Algorithms optimise for engagement, not coherence. They serve contradictory information, emotionally manipulative content, and conspiratorial narratives alongside credible journalism, not because they are designed to mislead, but because inconsistency generates clicks. A teenager immersed in this environment from age 12 onward is exposed to an informational landscape that actively punishes the building of stable predictive models, because any model is rapidly contradicted by the next piece of content. Under these conditions, the comprehensibility component of SOC cannot crystallise in the way Antonovsky envisaged.

Critically, this hypothesis explains four features of our data simultaneously. First, it explains the abruptness of the SOC decline (threshold effects produce step functions, not gradients). Second, it explains the variance compression (all adolescents are exposed to structurally similar algorithmic inconsistency, homogenising the input). Third, it explains the preservation of EI (emotional competencies develop in interpersonal micro-environments that algorithms do not control). Fourth, it explains the increase in EI in the 2024 cohort. Chronic exposure to emotionally charged content may paradoxically stimulate the development of emotional regulation as a survival mechanism, an "emotional immune response" to the algorithmic environment.

**3. The Cognitive Strategy Switch Hypothesis**

Third hypothesis connects the SOC–EI divergence to a distinction from computational neuroscience. The dual-system model of reinforcement learning (3, 4). Human decision-making relies on two parallel systems. The model-based system builds an internal model of the environment and uses it for forward planning. It is precise but slow and costly. The model-free system skips world-modelling entirely; it simply repeats actions that previously worked and avoids those that did not, fast and efficient, but rigid. The balance between these systems shifts with environmental conditions: stable environments reward model-based investment; volatile environments favour model-free processing (4).

We propose that SOC and EI align predominantly, though not exclusively, with these two systems. This is a simplification, EI includes components like "understanding emotions" that involve forward planning (11), and SOC may incorporate habitual coping routines. The claim concerns dominant processing mode, not a strict one-to-one mapping.

SOC resembles model-based processing. Comprehensibility corresponds to building a cognitive map of the world. Manageability corresponds to using that map for planning. Meaningfulness corresponds to the expected-value computation that justifies the investment in model-building. Antonovsky's observation that SOC crystallises in early adulthood (1) parallels model consolidation in reinforcement learning.

EI resembles model-free processing. It requires detecting emotional signals in real time and responding adaptively without a global world-model: positive emotional outcome → repeat; negative outcome → switch.

Applied to our data, this framework yields a coherent narrative. The 2014 cohort grew up in conditions stable enough to reward model-based investment. Some students built strong world-models, others less so — hence the wide SOC variance and the steep adaptation gradient. The 2015–2018 cohorts crossed a volatility threshold: the informational environment changed too fast for world-models to remain useful. The optimal strategy shifted toward model-free processing. SOC compressed; EI stabilised as the model-free system was still calibrating. The 2024 cohort, raised entirely in a volatile environment, shows the fully developed model-free profile: the highest EI scores and persistently low SOC.

This framework also explains the U-shaped trend in interpersonal EI. The 2014 cohort's high interpersonal EI was built on model-based social cognition, understanding others through mental models of their minds (theory of mind). The 2015–2018 cohorts occupy a transitional phase: model-based social cognition was breaking down, but model-free social processing (direct emotional signal detection) was not yet fully developed. The 2024 cohort achieves high interpersonal EI again, but on a different basis: real-time emotional tracking rather than mentalisation. Despite similar scores, the two cohorts likely rely on different mechanisms.

The implication for medical education is direct. If contemporary students have shifted toward model-free cognitive strategies, then educational approaches built on constructing coherent professional narratives, a fundamentally model-based endeavour — may be misaligned with how current learners actually process their environment. Professional Identity Formation as the construction of a "coherent professional self" (2) may need to be complemented by approaches that develop reactive expertise: rapid situation reading, flexible context-switching, and real-time emotional regulation under uncertainty.

**4. Integration and Converging Predictions**

The three hypotheses are not mutually exclusive; rather, they describe the same phenomenon at different levels of analysis. The reversed calibration hypothesis identifies the statistical mechanism (variance compression producing null correlations). The algorithmic comprehensibility threshold hypothesis identifies the environmental cause (the crossing of a digital ecology threshold around 2012). The cognitive strategy switch hypothesis identifies the cognitive process (adaptive rebalancing from model-based to model-free processing in response to environmental volatility).

**References**

1. Antonovsky A. Unraveling the Mystery of Health: How People Manage Stress and Stay Well. San Francisco: Jossey-Bass (1987).
2. Cruess RL, Cruess SR, Boudreau JD, Snell L, Steinert Y. A schematic representation of the professional identity formation and socialization of medical students and residents: a guide for medical educators. Acad Med (2015) 90:718-25. doi: 10.1097/ACM.0000000000000700
3. Daw ND, Niv Y, Dayan P. Uncertainty-based competition between prefrontal and dorsolateral striatal systems for behavioral control. Nat Neurosci (2005) 8:1704-11. doi: 10.1038/nn1560
4. Daw ND, Gershman SJ, Seymour B, Dayan P, Dolan RJ. Model-based influences on humans' choices and striatal prediction errors. Neuron (2011) 69:1204-15. doi: 10.1016/j.neuron.2011.02.027
5. Sackett PR, Yang H. Correction for range restriction: an expanded typology. J Appl Psychol (2000) 85:112-8. doi: 10.1037/0021-9010.85.1.112
6. Sackett PR, Lievens F, Berry CM, Landers RN. A cautionary note on the effects of range restriction on predictor intercorrelations. J Appl Psychol (2007) 92:538-44. doi: 10.1037/0021-9010.92.2.538
7. Twenge JM. iGen: Why Today's Super-Connected Kids Are Growing Up Less Rebellious, More Tolerant, Less Happy — and Completely Unprepared for Adulthood. New York: Atria Books (2017).
8. GUS [Statistics Poland]. Społeczeństwo informacyjne w Polsce. Wyniki badań statystycznych z lat 2012–2016 [Information society in Poland: statistical survey results 2012–2016]. Warsaw: GUS (2016). Available at: <https://stat.gov.pl/obszary-tematyczne/nauka-i-technika-spoleczenstwo-informacyjne/spoleczenstwo-informacyjne/>
9. Pew Research Center. Internet, smartphone and social media use in advanced economies. Washington, DC: Pew Research Center (2022). Available at: <https://www.pewresearch.org/global/2022/12/06/internet-smartphone-and-social-media-use-in-advanced-economies-2022/>
10. Eurostat. Being young in Europe today — digital world. Luxembourg: Publications Office of the European Union (2023). Available at: <https://ec.europa.eu/eurostat/statistics-explained/index.php/Being_young_in_Europe_today_-_digital_world>
11. Salovey P, Mayer JD. Emotional intelligence. Imagin Cogn Pers (1990) 9:185-211. doi: 10.2190/DUGG-P24E-52WK-6CDG
