# Supplementary material for: Different resources for different times: sense of coherence and emotional intelligence as correlates of adaptation in six cohorts of medical students
Source: Front Med (Lausanne). 2026 Jun 23;13:1860440. doi: 10.3389/fmed.2026.1860440 (PMC13337364; doi:10.3389/fmed.2026.1860440)
Supplement: Supplementary file 3 [file Table_3.DOCX]

**STROBE Statement - Checklist for Cross-Cohort Studies**

|  | No | Recommendation | Location in Manuscript |  |  |
| --- | --- | --- | --- | --- | --- |
| **Title and abstract** | 1 | (*a*) Indicate the study’s design with a commonly used term in the title or the abstract | Title: 'Sense of Coherence and Emotional Intelligence as Correlates of Adaptation in Six Cohorts  of Medical Students' - term 'Six Cohorts' and year span indicate the cross-cohort design. Abstract, Methods paragraph: 'We analysed data from six independent cross-cohorts…'. |  |  |
|  |  | (*b*) Provide in the abstract an informative and balanced summary of what was done and what was found | Abstract: four structured paragraphs (Background, Methods, Results, Conclusion) summarising rationale, design, key numerical findings, and the explicit epistemic limitation regarding restriction of range  in SOC. |  |  |
| Introduction | | |  |  |  |
| Background/rationale | 2 | Explain the scientific background and rationale for the investigation being reported | Section 1 Introduction - paragraphs 1–4 (rationale for studying SOC and EI as psychological resources  in medical education; literature gap regarding multi-cohort comparison of the two constructs). |  |  |
| Objectives | 3 | State specific objectives, including any prespecified hypotheses | Section 1 Introduction - final paragraph (aim statement and four pre-specified hypotheses H1–H4). |  |  |
| Methods | | |  |  |  |
| Study design | 4 | Present key elements of study design early in the paper | Section 2.1 Study design (cross-cohort comparison design, 2014–2018 and 2024 waves). Also stated in the Title, Abstract (Methods), and Section 5 Conclusion. |  |  |
| Setting | 5 | Describe the setting, locations, and relevant dates, including periods of recruitment, exposure, follow-up, and data collection | Section 2.1 Study design (setting: Medical University of Gdańsk, Poland; dates: 2014–2018 and 2024; justification of the 2020–2024 gap - COVID-19 pandemic and teaching stabilisation). Section 2.2 Participants (recruitment window April–May each year). Table 1 (per-cohort year of data collection  and sample sizes). |  |  |
| Participants | 6 | (*a*) Give the eligibility criteria, and the sources and methods of selection of participants | Section 2.2 Participants (inclusion criteria: second-year medical student status and fluency in Polish; exclusion criteria: insufficient Polish language proficiency or enrolment in a year other than the second  at the time of the study; recruitment through student group representatives and psychology classes; voluntary and anonymous participation). Table 1 (Eligible N and Response rate per cohort). |  |  |
| Variables | 7 | Clearly define all outcomes, exposures, predictors, potential confounders, and effect modifiers. Give diagnostic criteria, if applicable | Section 2.3 Measures - independent variables: Section 2.3.1 Sense of Coherence (SOC), Section 2.3.2 Emotional Intelligence (EI, total and intrapersonal/interpersonal subscales). Dependent variables: Section 2.3.3 (four indicators of adaptation - quality of life, current well-being, academic stress, satisfaction with medical studies). The absence of control for potential confounders (sex, socioeconomic status, place of residence) is explicitly acknowledged in Section 4.1 Limitations. |  |  |
| Data sources/ measurement | 8 | For each variable of interest, give sources of data and details of methods of assessment (measurement). Describe comparability of assessment methods if there is more than one group | Section 2.3.1 Sense of coherence - SOC-29 Life Orientation Questionnaire (Antonovsky, 1987); 7-point semantic differential; Polish version administered identically across all cohorts; Cronbach's  α = 0.82–0.95. Section 2.3.2 Emotional intelligence - INTE Polish adaptation (Jaworowska & Matczak, 2008) of the Schutte SSEIT (Schutte et al., 1998); 33 items, 5-point Likert; α = 0.87. Section 2.3.3 Dependent variables - author-administered single items (6-point QOL; 4-point well-being; 1–10 VAS stress; 1–10 satisfaction). Comparability across cohorts is discussed in Section 2.1 Study design and Section 4.1 Limitations. |  |  |
| Bias | 9 | Describe any efforts to address potential sources of bias | Section 2.2 Participants - voluntary and anonymous participation; data collection administered by individuals independent of the research team to minimise social desirability bias; all measurements conducted during April–May each year to control for phase of the academic year. Section 4.1 Limitations - discussion of potential selection bias in the 2024 cohort due to the lower response rate  (66% vs. 84–97% in 2014–2018). Section 4 Discussion, paragraph 1 - restriction-of-range effect  in 2015–2018 SOC data as an alternative explanation flagged up front. |  |  |
| Study size | 10 | Explain how the study size was arrived at | Section 2.2 Participants (N = 1,595 across six cohorts). Table 1 (per-cohort Eligible N, Response rate, and analytic n). No a priori formal power calculation was performed; the sample size reflects all eligible second-year students who consented in each data collection wave. The absence of a power analysis and the consequent limitations for the 2015–2018 cohorts (where SOC variance compression reduces statistical power) are acknowledged in Section 4.1 Limitations and Section 4 Discussion, paragraph 1. |  |  |
| Quantitative variables | 11 | Explain how quantitative variables were handled in the analyses. If applicable, describe which groupings were chosen and why | Section 2.3.3 Dependent variables - scale anchors and measurement units for each adaptation indicator. Section 2.4 Statistical analysis - adaptation indicators were treated as ordered categorical factors  in ANOVA; categories with fewer than 15 observations were excluded from the ANOVA analyses.  No continuous variable was dichotomised; SOC and EI total scores were treated as continuous outcomes in group comparisons. |  |  |
| Statistical methods | 12 | (*a*) Describe all statistical methods, including those used  to control for confounding | Section 2.4 Statistical analysis - between-group comparisons: Kruskal–Wallis with Dunn post-hoc and Benjamini–Hochberg correction. Interaction analyses: two-way Type III ANOVA with Games–Howell post-hoc. Significance level α = 0.05. All analyses performed in R version 4.4.1. Confounders were not statistically adjusted for; this single-predictor limitation is acknowledged in Section 4.1 Limitations. |  |  |
|  |  | (*b*) Describe any methods used to examine subgroups and interactions | Section 2.4 Statistical analysis - two-way ANOVA of cohort × adaptation indicator interactions for each of the four adaptation measures. Section 3 Results (Sections 3.1, 3.2, 3.3) - subgroup analyses by cohort reported throughout. Data Sheet 1 - Supplementary Tables S1–S28 provide full cell-level descriptive statistics and post-hoc comparisons for every subgroup. |  |  |
|  |  | (*c*) Explain how missing data were addressed | Section 2.2 Participants - note below Table 1 explicitly states that due to item-level missingness, effective sample sizes differ slightly across specific analyses (e.g., for EI analyses: 2016 n = 205, 2024  n = 262). No imputation was performed; analyses used complete cases per variable. Data Sheet 1 - Supplementary Table S11 reports per-cohort effective n for EI; Supplementary Tables S1, S4, S7 report effective n for SOC by adaptation category. |  |  |
|  |  | (*d*) If applicable, describe analytical methods taking account of sampling strategy | Not applicable. The study used a census approach within each cohort - all eligible second-year students were invited to participate in each data collection wave - rather than a complex sampling design. No sampling weights or design-based adjustments were therefore required. See Section 2.2 Participants. |  |  |
|  |  | (*e*) Describe any sensitivity analyses | No formal sensitivity analyses were performed in the present work. The absence of such analyses  - in particular, a re-analysis excluding the 2014 cohort, which disproportionately drives the cross-cohort SOC effect, and a measurement invariance test of the SOC-29 across cohorts - is explicitly acknowledged in Section 4 Discussion (paragraphs on restriction of range and measurement non-invariance) and Section 4.1 Limitations, and is recommended as a priority for follow-up work in Section 5 Conclusion. |  |  |
| Results | | |  |  |  |
| Participants | 13 | (a) Report numbers of individuals at each stage of study -eg numbers potentially eligible, examined for eligibility, confirmed eligible, included in the study, completing follow-up, and analysed | Section 2.2 Participants (overall N = 1,595 across six cohorts). Table 1 - per-cohort Eligible N, Response rate, and analytic n (2014: 297/337, 88%; 2015: 236/255, 92%; 2016: 214/252, 84%; 2017: 284/292, 97%; 2018: 270/310, 87%; 2024: 294/445, 66%). |  |  |
|  |  | (b) Give reasons for non-participation at each stage | Section 4.1 Limitations - discussion of the lower 2024 response rate, attributable to the expansion of the medical programme (445 eligible students in 2024 vs. 252–337 in earlier cohorts) with a comparable absolute number of respondents (n = 294). Individual-level reasons for non-participation are not available because recruitment was anonymous and voluntary (see Section 2.2 Participants). |  |  |
|  |  | (c) Consider use of a flow diagram | A separate flow diagram is not included. Table 1 provides the equivalent tabular summary of eligible, respondent, and analysed numbers per cohort. Additional missing-data details (variable-level effective n) are in Data Sheet 1 - Supplementary Tables S1, S4, S7, S11. |  |  |
| Descriptive data | 14 | (a) Give characteristics of study participants  (eg demographic, clinical, social) and information on exposures and potential confounders | Section 2.2 Participants - overall sample: 64% female; mean age 20.97, SD 1.52. Table 1 - per-cohort characteristics (n, female percentage, age M (SD)). Data on exposures (SOC and EI) and outcomes (adaptation indicators) are summarised per cohort in Data Sheet 1 - Supplementary Tables S1–S28. |  |  |
|  |  | (b) Indicate number of participants with missing data for each variable  of interest | Section 2.2 Participants (note below Table 1 on effective sample sizes differing slightly across analyses). Data Sheet 1 - Supplementary Table S11 reports effective n for total EI by cohort; Supplementary Tables S1, S4, S7 report effective n for SOC stratified by each adaptation category. Categories with n < 15 excluded from ANOVA (Section 2.4). |  |  |
| Outcome data | 15 | Report numbers of outcome events or summary measures | Section 3.1 Sense of coherence and adaptation (overall and cohort-wise medians and quartiles for SOC; Kruskal–Wallis and ANOVA statistics). Section 3.2 Emotional intelligence and adaptation (parallel summaries for total EI and intrapersonal/interpersonal subscales). Figures 1 and 2 (distributional summaries by cohort). Table 2 (overall and interaction p values for SOC vs. EI across four adaptation indicators). Data Sheet 1 - Supplementary Tables S1–S28 (full descriptive statistics and post-hoc comparisons). |  |  |
| Main results | 16 | (*a*) Give unadjusted estimates and, if applicable, confounder-adjusted estimates and their precision  (eg, 95% confidence interval). Make clear which confounders were adjusted for and why they were included | Section 3.1 and Section 3.2 - unadjusted mean differences with 95% confidence intervals reported for all significant post-hoc Games–Howell comparisons (e.g., SOC × QOL gradient in the 2014 cohort: 'wonderful' vs. 'not very good' = −48.8 points, 95% CI: −71.6 to −26.0, p < 0.0001). Figures 1, 2, 3 - graphical summaries of distributions and interaction patterns. Table 2 - overall and interaction p values. Data Sheet 1 - Supplementary Tables S3, S6, S9, S18, S20, S23 report all significant post-hoc comparisons with 95% CIs. Confounders were not statistically adjusted for; the resulting limitation  on interpretation is acknowledged in Section 4.1 Limitations. |  |  |
|  |  | (*b*) Report category boundaries when continuous variables were categorized | Section 2.3.3 Dependent variables - explicit scale anchors (QOL 6-point categorical scale from 'unhappy' to 'wonderful'; current well-being 4-point scale from 'unhappy' to 'very happy'; academic stress 1–10 VAS; satisfaction with medical studies 1–10). Section 2.4 Statistical analysis - ANOVA categories with  n < 15 were excluded from analysis. |  |  |
|  |  | (*c*) If relevant, consider translating estimates of relative risk into absolute risk for a meaningful time period | Not applicable. The present study does not estimate relative or absolute risks; outcomes are continuous/ordinal indicators of adaptation rather than dichotomous events, and the study design is not intended to yield incidence-based risk estimates. |  |  |
| Other analyses | 17 | Report other analyses done-eg analyses of subgroups and interactions, and sensitivity analyses | Section 3.1 - SOC × cohort interactions for all four adaptation indicators (QOL, well-being, academic stress, satisfaction). Section 3.2 - parallel analyses for total EI plus separate analyses of intrapersonal and interpersonal EI subscales. Section 3.3 - synthetic comparison of SOC and EI patterns (Tables 2 and 3). Data Sheet 1 - Supplementary Tables S1–S28 report the full set of subgroup and interaction analyses. Data Sheet 2 - Appendix 2 presents three alternative theoretical frameworks as speculative hypotheses  for future research (not as analyses of the present data). |  |  |
| Discussion | | |  |  |  |
| Key results | 18 | Summarise key results with reference to study objectives | Section 4 Discussion - first paragraph (summary of the principal observation: the pattern of associations between SOC, EI, and adaptation indicators differs across the six cohorts examined; SOC gradient confined to the 2014 cohort; EI gradients present in multiple cohorts; explicit restriction-of-range caveat). Section 5 Conclusion - concise restatement in epistemically hedged form. |  |  |
| Limitations | 19 | Discuss limitations of the study, taking into account sources of potential bias or imprecision. Discuss both direction and magnitude of any potential bias | Section 4.1 Limitations - cross-cohort design does not permit causal inference or unambiguous separation of cohort from period effects; cohort effects confounded with historical events (COVID-19 pandemic, curricular changes, war in Ukraine); no control for confounders (sex, socioeconomic status, place of residence); lower response rate in the 2024 cohort raises potential selection bias. Section 4 Discussion, paragraph 1 - the most important limitation (restriction of range in SOC variance in 2015–2018) is integrated into the first paragraph of the Discussion itself rather than deferred, so that it frames all subsequent interpretation. |  |  |
| Interpretation | 20 | Give a cautious overall interpretation of results considering objectives, limitations, multiplicity of analyses, results from similar studies, and other relevant evidence | Section 4 Discussion - all paragraphs framed as observations that differ across cohorts rather than evidence of generational change; interpretation is explicitly conditional on future measurement invariance testing of the SOC-29 and replication in independent institutions (paragraph 2). Section 5 Conclusion - epistemically hedged wording ('compatible with, but do not establish') and explicit call for follow-up analyses. The discussion of multiplicity of tests and convergence with prior literature  (Tartas et al., 2011, 2014; Boudreau et al., 2024) appears throughout Section 4. |  |  |
| Generalisability | 21 | Discuss the generalisability (external validity) of the study results | Section 4.1 Limitations - single-institution design at the Medical University of Gdańsk limits external validity; cohort effects cannot be disentangled from institution-specific changes (curricular reforms, admissions criteria, class culture). Section 5 Conclusion - explicit recommendation for replication  in independent institutions before any generalisation to other populations of medical students or other generations is warranted. |  |  |
| Other information | | |  |  |  |
| Funding | 22 | Give the source of funding and the role of the funders for the present study and, if applicable, for the original study on which the present article is based | Declarations - Funding section (grant ST-50#2018, 'Psychological factors determining quality of life  in selected clinical and non-clinical groups', funded by the Medical University of Gdańsk, Poland).  The funder had no role in study design, data collection, analysis, interpretation, or manuscript preparation. |  |  |
